# Generalisable prediction models for outcomes after lumbar spinal stenosis surgery: a model development and external validation study

**DOI:** 10.1016/j.eclinm.2026.103989

**Published:** 2026-05-28

**Authors:** Bjørnar Berg, Allan Abbott, Casper Friis Pedersen, Henrik Hedevik, Martin A. Gorosito, Henrik Lykke Joakimsen, Per Joel Burman, Karl Øyvind Mikalsen, Mikkel Østerheden Andersen, Tor Ingebrigtsen, Tore Solberg, Margreth Grotle

**Affiliations:** a Centre for Intelligent Musculoskeletal Health, Faculty of Health Sciences, Oslo Metropolitan University, Oslo, Norway; b Unit of Physiotherapy, Division of Prevention, Rehabilitation and Community Medicine, Department of Health, Medicine and Caring Sciences, Linköping, Sweden; c Clinical Department of Orthopaedics in Linköping, Region Östergötland, Linköping, Sweden; d Center for Spine Surgery and Research, Spine Center of Southern Denmark, Lillebaelt Hospital, University of Southern Denmark, Kolding, Denmark; e Department of Computer Science, Oslo Metropolitan University, Oslo, Norway; f Department of Clinical Medicine, Faculty of Health Sciences, UiT The Arctic University of Norway, Tromsø, Norway; g The Norwegian Centre for Clinical Artificial Intelligence, University Hospital of North Norway, Tromsø, Norway; h UiT Machine Learning Group, Department of Physics and Technology, UiT the Arctic University of Norway, Tromso, Norway; i Department of Neurosurgery and The Norwegian Registry for Spine Surgery, The University Hospital of North Norway, Tromsø, Norway; j Division of Clinical Neuroscience, Department of Research and Innovation, Oslo University Hospital, Oslo, Norway

**Keywords:** Lumbar spinal stenosis, Spine surgery, Prediction model, Machine learning, External validation

## Abstract

**Background:**

Lumbar spinal stenosis is a leading indication for spine surgery, but outcomes are heterogeneous. We aimed to develop and externally validate prediction models for 12-month disability and pain to inform shared decision-making.

**Methods:**

This registry-based multicentre cohort study used data from three national spine registries of patients (≥16 years) undergoing elective lumbar spinal stenosis surgery. Data from the Norwegian Registry for Spine Surgery (NORspine, 2007–2023) were used for model development and internal-external cross-validation (IECV). External validation was carried out in the Swedish Registry (SweSpine, 2016–2022) and Danish Registry (DaneSpine, 2009–2022) with data collected by the Spine Centre of Southern Denmark. The primary outcome was the Oswestry Disability Index (ODI) at 12 months, modelled as a continuous and binary measure (acceptable symptom state). Secondary outcomes were Numeric Rating Scale (NRS) back and leg pain at 12 months. Logistic regression, linear regression, and XGBoost models were applied with 16 predictors. Missing data were handled using multiple imputation. Performance was assessed by calibration, mean absolute error (MAE), adjusted R^2^, and C-statistics. This study is registered with Open Science Framework (https://osf.io/qz27b/).

**Findings:**

The development cohort included 31,908 patients (52.4% female, 47.6% male). The external validation cohorts included 30,700 from SweSpine (52.8% female, 47.2% male) and 4063 from DaneSpine (54.6% female, 45.4% male). Twelve-month outcome completeness was 77% in the development cohort and ranged from 66% to 80% across the external validation cohorts. For ODI, linear regression achieved a pooled MAE of 12.4 (95% CI 11.8–13.1) after IECV, and 13.3 (95% CI 13.2–13.4) and 12.3 (95% CI 12.0–12.7) at external validation. Adjusted R^2^ values ranged from 0.26 to 0.33. Calibration was acceptable, with slopes near 1 and calibration-in-the-large ranging from −0.47 after IECV to 1.28–1.54 at external validation, indicating minor systematic underprediction. The binary ODI model achieved C-statistics of 0.75 (95% CI 0.74–0.76) after IECV, and 0.78 (95% CI 0.78–0.79) and 0.76 (95% CI 0.74–0.77) at external validation. Pain models showed lower performance (MAE 2.2–2.6; C-statistics 0.64–0.73). XGBoost yielded similar results.

**Interpretation:**

Models predicting disability and pain were well calibrated and generalisable across Scandinavian countries, with the best overall performance for disability. These findings provide a foundation for prospective evaluation in future studies to determine the impact on decision-making and patient outcomes in clinical practice.

**Funding:**

Research Council of Norway.

**Research in context**

**Evidence before this study**

A PubMed search was conducted from inception to February 15, 2026, using the terms (lumbar spine OR spinal stenosis) AND (prediction model OR prognostic model OR machine learning) AND (disability OR pain), with no language restriction. Several studies have developed models predicting disability or pain after lumbar spinal stenosis surgery, but most had modest sample sizes and lacked external validation or focused on fusion procedures. All previously externally validated models dichotomised outcomes and generally showed acceptable discrimination but poor calibration, limiting their potential for prospective evaluation and future clinical use.

**Added value of this study**

Using data from 66,671 patients across three national spine registries in Norway, Sweden, and Denmark, this study presents generalisable statistical and machine learning models predicting 12-month disability and pain after lumbar spinal stenosis surgery. Based on 16 routinely collected predictors, separate models for continuous and binary outcome definitions were rigorously evaluated using internal-external cross-validation within Norway and external validation across countries. The models demonstrated good and consistent performance across care settings, with the best overall performance for disability.

**Implications of all the available evidence**

Prediction modelling in spine surgery has largely remained at the development stage. This study demonstrates that models based on routinely collected clinical information can generalise across national registries and care settings, providing a foundation for prospective evaluation in future studies and potential clinical integration to support shared decision-making.

## Introduction

Lumbar spinal stenosis is a major cause of disability and pain,^1^ and the most common indication for spine surgery among older adults.^2^ Surgical intervention aims to alleviate symptoms and improve function when conservative treatment fails.^3^ However, postoperative outcomes vary widely, with some patients experiencing persistent disability and pain.^4^ Surgical decisions typically rely on clinical judgment, but prediction of postoperative outcomes at the individual level remains challenging.^5^ Given the elective nature of most lumbar spinal stenosis surgeries,^6^ shared decision-making is essential to ensure that patients have realistic expectations and that surgical interventions provide meaningful benefit.^7^

Accurate prediction of postoperative disability and pain could enhance decision-making by enabling personalised risk assessment. However, the heterogeneity of lumbar spinal stenosis makes outcome prediction challenging.^3^ Several prediction models have been developed for various postoperative outcomes, with varying performance.^8^, ^9^, ^10^, ^11^, ^12^, ^13^, ^14^ While some binary prediction models have demonstrated relatively robust discrimination at external validation, their calibration has only been fair, raising concerns about their application in clinical practice.^15^, ^16^, ^17^, ^18^

Given the variability in postoperative outcomes and uncertainty in patient selection, accurate and generalisable prediction models are needed to improve surgical decision-making and ensure reliable clinical application. Such models are intended to be deployed in decision support tools and inform preoperative counselling and shared decision-making among clinicians and patients being considered for surgery in specialist healthcare. We build on previous work evaluating the generalisability of prediction models for lumbar disc herniation surgery^19^^,^^20^ and complement Norwegian algorithm-development approved for clinical implementation, which uses a broader set of predictors not consistently available in other national spine registries.^21^ Accordingly, this study aimed to develop and externally validate multivariable statistical and machine learning models predicting disability and pain following lumbar spinal stenosis surgery, using data from national spine registries in Norway, Sweden, and Denmark.

## Methods

### Design

This registry-based multicentre cohort study used prospectively collected data from three national spine registries including patients who underwent elective lumbar spinal stenosis surgery. The methodology followed the framework proposed by the Prognosis Research Strategy (PROGRESS) group,^22^ and the study was reported in accordance with the Transparent Reporting of a multivariable prediction model for Individual Prognosis or Diagnosis (TRIPOD + AI) guideline.^23^ The protocol was prospectively registered on Open Science Framework (https://osf.io/qz27b/).

### Ethics

This study is part of the AID-Spine project and received ethical approval from the Regional Ethics Committee of the Health Region of South-East Norway (2022/371282). The Norwegian Registry for Spine Surgery (NORspine) protocol was approved by the Data Protection Authority of Norway. Approval for the use of anonymised data from the Swedish Registry for Spine Surgery (SweSpine) was granted by the Regional Ethics Committee in Linköping, Sweden (2021–04914). In Denmark, the use of anonymised data from the Spine Centre of Southern Denmark, registered in the Danish Registry for Spine Surgery (DaneSpine), is exempt from ethical review under Danish law. All patients in the registries have been informed and have consented for the use of their data in research and quality improvement.

### Data source and patient population

Data from the NORspine^24^ were used for model development and internal-external cross-validation, while external validation was conducted using data from SweSpine and DaneSpine.^25^^,^^26^ NORspine was selected as the development cohort, as it is the setting for an ongoing implementation initiative aimed at deploying prediction models in a decision support tool integrated in the electronic health record.^21^ Currently, NORspine covers all 42 surgical units performing spine surgery in Norway, with a capture rate of 81%.^27^ Patients who underwent surgery between January 1, 2007, and December 01, 2023, were included. SweSpine currently covers 46 of 47 surgical centres performing spine surgery in Sweden, with a capture rate of 86%.^25^ Data from surgeries performed between January 1, 2016, and December 31, 2022, were included. DaneSpine provided data from patients treated at the Spine Centre of Southern Denmark between August 1, 2009, and December 31, 2022, with a current regional capture rate of 96%.^28^ In all three registries, the capture rate is defined as the proportion of eligible spine surgeries recorded in the registry relative to those identified in national administrative data. Capture rate analyses are conducted by national registry authorities, and the administrative data are considered nearly complete, as reporting is mandatory for reimbursement. Eligible patients in all cohorts were defined using the same criteria: adults aged 16 years and older undergoing elective decompression surgery (with or without fusion) for lumbar spinal stenosis. Reoperations within 90 days are classified as complications in the registries, with no new case registration, but repeat surgeries performed more than 90 days after the index surgery were included as new cases in the current study.

All three registries adhere to similar, standardized data collection procedures.^29^ Before surgery, patients complete a preoperative questionnaire that includes patient-reported outcomes at the time of surgical admission (baseline). Surgeons document diagnosis, imaging findings, and comorbidities using a standardized form. At the 12-month follow-up, patients complete questionnaires reassessing the same patient-reported outcomes collected preoperatively.

### Outcomes

The primary outcome was the Oswestry Disability Index (ODI) at the 12-month follow-up. The ODI is a 10-item score ranging from 0 to 100, with higher scores indicating greater back-pain related disability.^30^ The score was modelled both as a continuous outcome and as a binary outcome, using a cut-off of 22 points to indicate an acceptable symptom state.^31^ Secondary outcomes included back and leg pain intensity, assessed using the Numeric Rating Scale (NRS, range 0–10, with higher scores indicating greater pain). These were modelled both continuously and as binary outcomes, with a cut-off of 3 points to reflect an acceptable pain level following spine surgery.^32^ We chose an acceptable symptom state as our binary outcome rather than a change-based measure. Both approaches have strengths and limitations in defining treatment success. A state-attainment criterion may be less dependent on baseline severity and reflects whether a patient feels well at follow-up, which has been suggested to be particularly meaningful to patients.^32^

### Predictors

Predictor variables were selected based on distribution (missingness and variability) and their availability across the three registries. Variables with more than 50% missing data in any of the registries were excluded. The remaining predictors were then harmonized to ensure equivalent definitions across cohorts (see Supplementary Table S1 for variable mapping). All predictors were measured prior to surgery. A total of 16 predictors were included, covering demographics, clinical characteristics, comorbidities, work-related factors, analgesic use, and prior lumbar spine surgery.

### Sample size

The sample size was determined by the number of surgeries recorded in the three registries during the study periods. Sample size calculations were performed to confirm adequacy for model development and validation.^33^^,^^34^ For model development, a minimum of 775 surgical cases were required for the continuous ODI score model, based on 33 predictor parameters (to allow for all predictors plus transformations), assuming an R^2^ of 0.30, a mean ODI score of 23.3, and a standard deviation of 18.2. For binary outcomes, a minimum of 1558 cases (with 733 events) were required, assuming an outcome prevalence of 47% (not experiencing an acceptable symptom state) and a C-statistic of 0.74.^9^

For external validation, using input values from the developed models and conservatively assumed perfect calibration, a minimum of 990 cases was required for the continuous ODI model for precise estimation of calibration and explained variance. For binary outcomes, calculation based on a normally distributed linear predictor (mean = 0.18, SD = 1.13) indicated that at least 2340 cases (1100 events) were required for precise estimation of calibration and discrimination. Additional details of the sample size calculations are described in the Supplementary Method.

The number of cases available in both the development and validation cohorts far exceeds the minimum required sample size estimates for regression-based prediction models.

### Data cleaning and quality checks

All three registries undergo periodic quality assessments by the respective registry owners. Additional data-quality checks were conducted during data cleaning, including identifying duplicate entries and outliers. Systematically missing variables or variable categories across cohorts were assessed during predictor selection and harmonization.

### Statistical analysis

Eligible patients were identified based on registry-recorded classification of surgery using procedure and diagnostic codes or indication for surgery recorded by the surgeon, with age defined from date of birth relative to the date of surgery. Descriptive characteristics were summarized separately for the development and validation cohorts. Missing values in predictors and outcomes were addressed with multiple imputation by chained equations, using logistic regression for binary variables, predictive mean matching for continuous variables, and ordered or multinomial logistic regression for categorical variables.^35^ Data were assumed to be missing at random. Forty imputed datasets were generated using 10 iterations to ensure stable and reliable estimates, based on the proportion of missing data. All predictors and outcomes were included in the imputation models. The total score was imputed for questionnaire-based variables. For the development data, imputations were performed separately for each health region to allow the distribution of imputed values to differ. Validation cohorts were imputed separately to prevent data leakage. Model performance was assessed in each imputed dataset, and results were aggregated using Rubin`s rule.^36^ The same model performance estimates, with 95% confidence intervals (CI), were evaluated at both the development and validation stages. For continuous models, we report adjusted R^2^ (proportion of explained variance), mean absolute error (the average absolute difference between predicted and observed values), calibration-in-the-large (CITL; 0 indicates perfect calibration), and calibration slope (1 indicates perfect calibration). For binary models, we report discrimination (C-statistic; 0.5 indicates no discrimination, 1 indicates perfect discrimination) and calibration (CITL and slope).

#### Model development and internal-external cross-validation

We utilized two modelling approaches for both continuous and binary outcomes: regression-based models (linear and logistic) and a machine learning model (XGBoost). To account for potential non-linear relationships, multivariable fractional polynomials were applied within the regression models.^37^ Clustering of patients within individual surgical centres was accounted for using clustered standard errors to address potential intra-centre correlation. For XGBoost, Bayesian optimisation was used for hyperparameter tuning, nested within each iteration of the internal-external cross-validation to avoid data leakage.

Internal-external cross-validation was performed to obtain a more realistic estimate of model performance and evaluate generalisability across geographical regions.^38^ In this approach, data were partitioned into clusters based on Norway’s four Regional Health Authorities, with an additional cluster for private hospitals. In each iteration, four clusters were used for model development and the remaining cluster for validation, rotating through all clusters across five cycles. Model performance was evaluated within each region, and overall performance metrics were pooled across clusters using a random-effects meta-analysis with Hartung-Knapp-Sidik-Jonkman variance correction.^39^^,^^40^ The internal-external cross-validation approach is illustrated in Supplementary Fig. S1.

To explore potential variations in model performance by sex and among patients who underwent fusion surgery, performance metrics were also computed separately for these subgroups using individual-level predictions. Sensitivity analyses based on complete cases were also conducted to assess the robustness of the main analyses. All analyses were prespecified in the study protocol.

#### Model validation

The final regression and XGBoost models were trained on the full development cohort, including re-optimisation of hyperparameters for XGBoost, and were subsequently applied to the external validation cohorts without further re-fitting to evaluate their performance. The fractional polynomial specifications and XGBoost hyperparameters identified in the development cohort were applied unchanged. In addition to model performance metrics with 95% confidence intervals, calibration plots comparing observed versus predicted outcomes or probabilities are presented. For binary models, we also present risk distribution plots showing the predicted probability distribution for each outcome category, and decision curve analyses to evaluate clinical utility across decision thresholds.^41^^,^^42^

Stata Version 18.0 was used for data cleaning, multiple imputation, and the traditional statistical models. The machine learning algorithms were implemented using R 4.0.1. Analysis code is available in repository https://github.com/bjornarb88/AID-Spine-LSS-models.

### Patient involvement

Aims and designs of the study were discussed with a patient representative who regularly attends research meetings as part of the AID-Spine project.

### Role of the funding source

The funder of the study (Research Council of Norway) had no involvement in study design, data collection, data analyses, data interpretation, or the writing of the report.

## Results

**Fig. 1:**
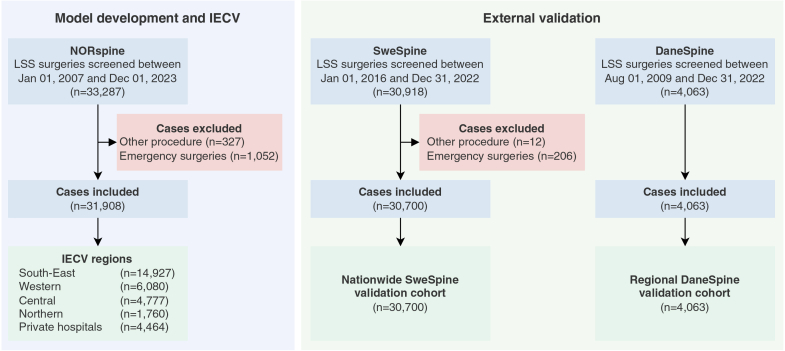
Study profile. IECV = internal-external cross-validation; LSS = lumbar spinal stenosis.

Of 33,287 lumbar spinal stenosis surgeries screened in NORspine, 31,908 were included for model development and internal-external cross-validation (Fig. 1). The SweSpine and DaneSpine registries contributed 30,700 and 4063 cases for external validation, respectively. Baseline characteristics for all cohorts are presented in Table 1, and the extent of missing values for predictors and outcomes is shown in Supplementary Table S2. Twelve-month outcome completeness was 77% in the development cohort and ranged from 66% to 80% across the external validation cohorts.

**Table 1:** Descriptive statistics for model development and validation cohorts.

	NORspine (development) (n = 31,908)	SweSpine (validation) (n = 30,700)	DaneSpine (validation) (n = 4063)
Sex			
Male	15,191 (47.6)	14,488 (47.2)	1845 (45.4)
Female	16,717 (52.4)	16.212 (52.8)	2.218 (54.6)
Age (years)	65.4 ± 11.9	67.3 ± 11.0	67.4 ± 11.4
Body mass index (kg/m^2^)	27.7 ± 4.5	27.7 ± 4.2	27.2 ± 4.1
Smoker	5678 (18.0)	1569 (5.2)	889 (22.4)
Work status:			
Working/student	5344 (17.0)	6772 (22.1)	365 (9.0)
Age retirement	15,809 (50.1)	18,551 (60.6)	2554 (62.9)
Sick leave	4703 (14.9)	4328 (14.1)	620 (15.3)
Welfare benefits^a^	5678 (18.0)	970 (3.2)	524 (12.9)
Back pain:			
Less than 3 months	1110 (3.6)	1982 (6.5)	438 (11.0)
3–12 months	6264 (20.5)	6302 (20.8)	1015 (25.5)
12–24 months	6746 (22.1)	6608 (21.8)	724 (18.2)
More than 24 months	16,379 (53.7)	15,450 (50.9)	1801 (45.3)
Leg pain:			
Less than 3 months	2010 (6.7)	1872 (6.2)	346 (8.7)
3–12 months	8578 (28.6)	8821 (29.0)	1509 (37.9)
12–24 months	7757 (25.9)	8061 (26.5)	952 (23.9)
More than 24 months	11,605 (38.8)	11,681 (38.4)	1178 (29.5)
ODI	39.5 ± 15.1	43.1 ± 16.0	42.2 ± 15.6
NRS back pain	6.6 ± 2.2	6.1 ± 2.6	5.5 ± 2.8
NRS leg pain	6.6 ± 2.2	6.8 ± 2.3	6.6 ± 2.4
EQ5D	0.57 ± 0.20	0.56 ± 0.18	0.39 ± 0.31
Health status (EQ-VAS)^b^	48.9 ± 20.1	48.5 ± 22.2	50.3 ± 23.5
Anxiety or depression^c^	9124 (29.6)	16,753 (55.5)	601 (15.1)
Previous surgery:			
Zero	23,073 (72.7)	24,165 (79.3)	2.860 (71.7)
One	6199 (19.5)	4551 (14.9)	710 (17.8)
Two or more	2446 (7.7)	1743 (5.7)	418 (10.5)
Comorbidities:			
None	11,919 (37.4)	22,627 (75.2)	3040 (76.8)
One	9871 (30.9)	6101 (20.3)	777 (19.6)
Two	6240 (19.6)	1189 (4.0)	121 (3.1)
Three or more	3878 (12.2)	160 (0.5)	19 (0.5)
Analgesics frequency:			
Monthly	8372 (26.7)	4834 (15.9)	843 (24.6)
Weekly	4861 (15.5)	9075 (29.9)	2580 (75.4)^d^
Daily	18,098 (57.8)	16,451 (54.1)	–

Values are numbers (%) or mean ± standard deviation.

ODI = Oswestry Disability Index (range 0–100); NRS = Numeric Rating Scale (range 0–10).

a Full or partial disability pension or work assessment allowance.

b EQ-5D Visual Analogue Scale (range 0–100).

c EQ-5D questionnaire; 5th item, moderate to severe (3 L) or moderate to extreme (5 L).

d Weekly or daily.

**Fig. 2:**
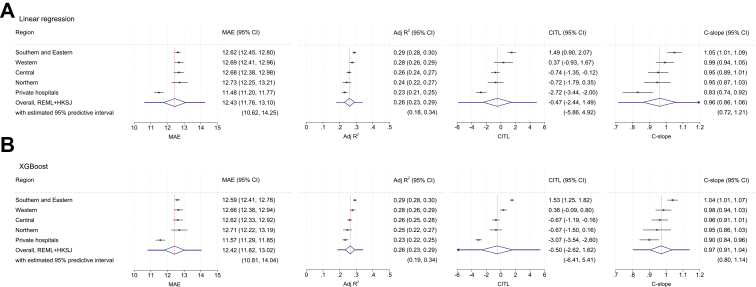
Internal-external cross-validation within the development cohort across five geographical regions and the overall estimation across regions for the Oswestry Disability Index continuous model from (A) Linear regression and (B) XGBoost. MAE = mean absolute error; Adj R2 = Adjusted R^2^ CITL = calibration-in-the-large; C-slope = calibration slope; REML + HKSJ = random-effects meta-analysis with Hartung-Knapp-Sidik-Jonkman variance correction.

For the ODI linear regression model, pooled estimates from the random effects meta-analysis showed a mean absolute error of 12.4 (95% CI 11.8 to 13.1) and an adjusted R^2^ of 0.26 (95% CI 0.23 to 0.29), with minor regional heterogeneity (Fig. 2A). The model was well-calibrated overall (pooled estimates: CITL −0.47 [95% CI −2.44, 1.49]; calibration slope 0.96 [95% CI 0.86, 1.06]). Regional CITL values ranged from 1.49 to −2.72, and calibration slope from 1.05 to 0.83 (Fig. 2A). Region-specific calibration plots are provided in Supplementary Fig. S6. Model coefficients, including the intercept and all fractional polynomial transformations, are provided in Supplementary Table S3. The selected hyperparameter configuration for the XGBoost model is shown in Supplementary Table S4. XGBoost achieved performance similar to the linear regression model (Fig. 2B).

Performance for pain outcomes was somewhat lower than for the ODI models. The pooled adjusted R^2^ from linear regression was 0.14 (95% CI 0.11 to 0.16) for NRS back pain (Supplementary Fig. S2A) and 0.10 (95% CI 0.07 to 0.12) for NRS leg pain (Supplementary Fig. S3A). Both models were well-calibrated overall, and there were no appreciable differences between the modelling approaches (Supplementary Fig. S2B and S3B).

**Fig. 3:**
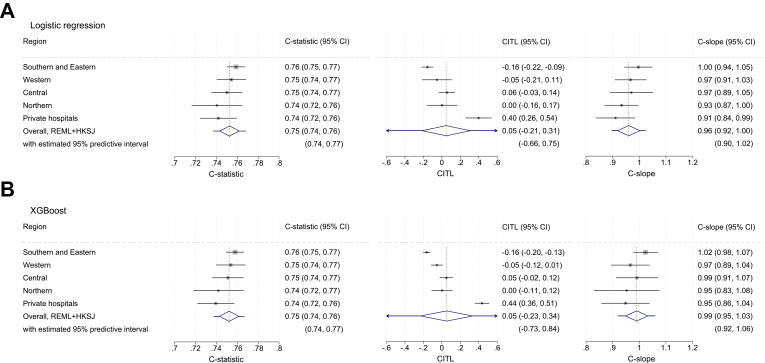
Internal-external cross-validation within the development cohort across five geographical regions and the overall estimation across regions for the Oswestry Disability Index binary model from (A) Logistic regression and (B) XGBoost. CITL = calibration-in-the-large; C-slope = calibration slope; REML + HKSJ = random-effects meta-analysis with Hartung-Knapp-Sidik-Jonkman variance correction.

The proportion of patients experiencing an acceptable symptom state for ODI was 54% overall. The proportions were similar across the four public health regions (50%–52%) but higher among patients operated in private hospitals (70%). For the logistic regression model, the pooled C-statistic from random effects meta-analysis was 0.75 (95% CI 0.74 to 0.76) for ODI (Fig. 3A). The model showed no evidence of miscalibration, with a pooled CITL of 0.05 (95% CI −0.21 to 0.31) and a calibration slope of 0.96 (95% CI 0.92 to 1.00). Only minor regional heterogeneity was observed, except for some underestimation of the predicted probability of experiencing an acceptable symptom state in private hospitals (CITL 0.40, 95% CI 0.26 to 0.54) (Fig. 3A). Region-specific calibration plots are provided in Supplementary Fig. S7. Model coefficients, including the intercept and all transformation terms, are presented in Supplementary Table S5. Similar performance was found for XGBoost both in terms of discrimination and calibration (Fig. 3B).

For pain outcomes, 48% of patients experienced an acceptable symptom state for NRS back pain and 55% for NRS leg pain. Across the four public health regions, proportions were similar (45%–46% for back pain and 52%–53% for leg pain) but higher in private hospitals (62% and 67%, respectively). Logistic regression models achieved pooled C-statistics of 0.69 (95% CI 0.68 to 0.70) for NRS back pain (Supplementary Fig. S4A) and 0.66 (95% CI 0.65 to 0.68) for NRS leg pain (Supplementary Fig. S5A). Both models were well calibrated overall, and performance metrics were comparable for XGBoost (Supplementary Figs. S4B and 5B).

**Fig. 4:**
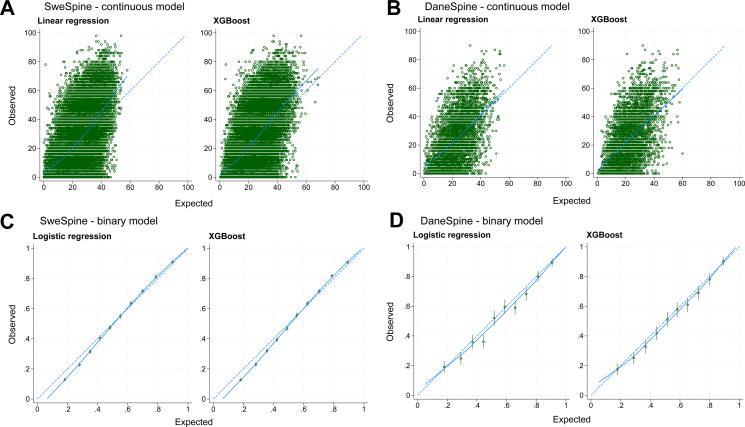
Model calibration in external validation data for the Oswestry Disability Index, with continuous models in (A) SweSpine and (B) DaneSpine, and binary models in (C) SweSpine and (D) DaneSpine. The dashed blue line indicates perfect calibration. The solid blue line is a fitted Loess smoother curve for the predicted values or probabilities. Points represent individual observations (continuous models) or grouped observed proportions (binary models), with vertical lines indicating 95% confidence intervals.

External validation in SweSpine and DaneSpine demonstrated model performance consistent with the development phase for ODI (Table 2). In SweSpine, the mean absolute error was 13.3 (95% CI 13.2 to 13.4) for linear regression. In DaneSpine, the corresponding value was 12.3 (95% CI 12.0 to 12.7). Explained variance was slightly higher at external validation, with adjusted R^2^ values of 0.33 (95% CI 0.33 to 0.34) in SweSpine and 0.28 (95% CI 0.26 to 0.30) in DaneSpine. Calibration was acceptable, with slopes close to 1 indicating appropriate spread of predicted values (Fig. 4). CITL values suggested minor systematic underprediction (i.e., predicted ODI values were lower than observed) in SweSpine (1.28 [95% CI 0.98 to 1.57]) for linear regression. Similar calibration performance was observed in DaneSpine (Table 2). Similar performance was found for XGBoost (Supplementary Table S6).

**Table 2:** Performance metrics with 95% confidence intervals of traditional statistical models.

	NORspine (n = 31,908)^a^	SweSpine (n = 30,700)	DaneSpine (n = 4063)
Continuous models			
ODI			
MAE	12.4 (11.8, 13.1)	13.3 (13.2, 13.4)	12.3 (12.0, 12.7)
Adjusted R^2^	0.26 (0.23, 0.29)	0.33 (0.33, 0.34)	0.28 (0.26, 0.30)
CITL	−0.47 (−2.44, 1.49)	1.28 (0.98, 1.57)	1.54 (0.95, 2.13)
C-slope	0.96 (0.86, 1.06)	1.17 (1.14, 1.20)	0.94 (0.89, 1.00)
NRS back pain			
MAE	2.16 (2.15, 2.18)	2.21 (2.19, 2.23)	2.25 (2.21, 2.31)
Adjusted R^2^	0.14 (0.11, 0.16)	0.21 (0.20, 0.22)	0.19 (0.18, 0.21)
CITL	−0.04 (−0.31, 0.22)	−0.11 (−0.16, −0.06)	−0.27 (−0.36, −0.16)
C-slope	0.96 (0.91, 1.02)	1.15 (1.12, 1.18)	1.04 (0.96, 1.12)
NRS leg pain			
MAE	2.36 (2.28, 2.43)	2.51 (2.49, 2.53)	2.59 (2.54, 2.65)
Adjusted R^2^	0.10 (0.07, 0.12)	0.13 (0.13, 0.14)	0.07 (0.06, 0.08)
CITL	−0.04 (−0.31, 0.23)	0.01 (−0.07, 0.09)	0.17 (0.05, 0.28)
C-slope	0.94 (0.83, 1.04)	1.16 (1.12, 1.21)	0.97 (0.84, 1.09)
Binary models			
ODI			
C-statistic	0.75 (0.74, 0.76)	0.78 (0.78, 0.79)	0.76 (0.74, 0.77)
CITL	0.05 (−0.21, 0.31)	−0.06 (−0.10, −0.03)	−0.15 (−0.24, −0.06)
C-slope	0.96 (0.92, 1.00)	1.16 (1.13, 1.20)	0.97 (0.88, 1.06)
NRS back pain			
C-statistic	0.69 (0.68, 0.70)	0.73 (0.72, 0.74)	0.73 (0.71, 0.75)
CITL	0.03 (−0.18, 0.23)	0.13 (0.08, 0.17)	0.18 (0.11, 0.27)
C-slope	0.96 (0.91, 1.01)	1.17 (1.12, 1.22)	1.06 (0.95, 1.17)
NRS leg pain			
C-statistic	0.66 (0.65, 0.68)	0.68 (0.68, 0.69)	0.64 (0.62, 0.66)
CITL	0.02 (−0.17, 0.21)	0.09 (0.04, 0.13)	−0.10 (−0.18, −0.01)
C-slope	0.95 (0.87, 1.02)	1.12 (1.06, 1.18)	0.86 (0.72, 1.00)

ODI = Oswestry Disability Index; NRS = Numeric Rating Scale; MAE = mean absolute error; CITL = calibration-in-the-large; C-slope = calibration slope.

a Internal-external cross-validation estimates pooled across regions using random-effects meta-analysis.

Model performance for NRS back and leg pain was slightly lower, consistent with performance during internal-external cross-validation (Table 2). For NRS back pain, the mean absolute error from linear regression was 2.21 (95% CI 2.19 to 2.23) in SweSpine and 2.25 (95% CI 2.21 to 2.31) in DaneSpine. The corresponding values for NRS leg pain were 2.51 (95% CI 2.49 to 2.53) and 2.59 (95% CI 2.54 to 2.65). Models for both outcomes were well-calibrated, and comparable results were observed for XGBoost (Supplementary Table S6 and Supplementary Figs. S6 and S7).

In both SweSpine and DaneSpine, 53% of patients experienced an acceptable symptom state for ODI. External validation of the ODI model showed slightly improved discrimination compared with internal-external cross-validation, with C-statistics from logistic regression of 0.78 (95% 0.78 to 0.79) in SweSpine and 0.76 (95% CI 0.74 to 0.77) in DaneSpine. For NRS back and leg pain, the proportions experiencing an acceptable symptom state were 56% for both outcomes in SweSpine, and 60% and 57% in DaneSpine. C-statistics ranged from 0.73 (95% CI 0.72 to 0.74) to 0.64 (95% CI 0.62 to 0.66) (Table 2). There were no major signs of miscalibration observed for either outcome or modelling approach, although the NRS back pain models showed minor underprediction in both cohorts (Table 2 and Supplementary Figs. S6 and S7).

**Fig. 5:**
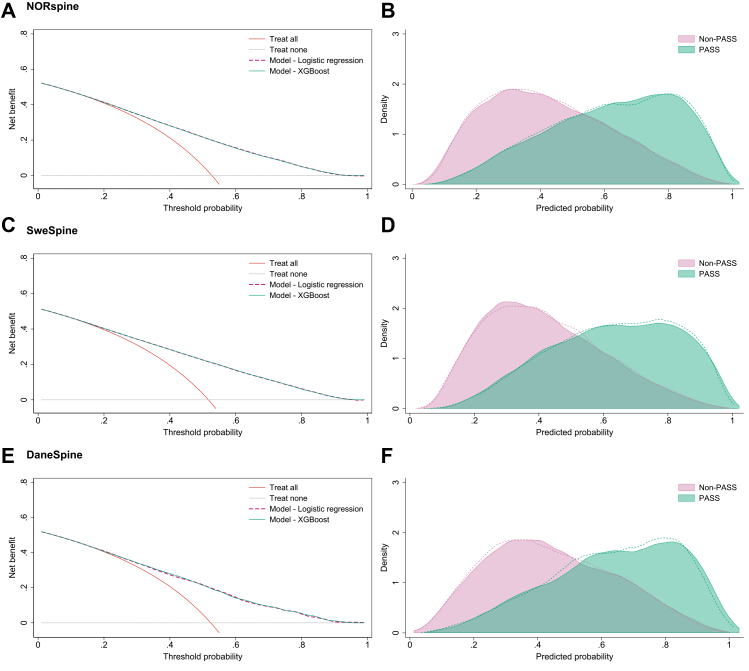
Decision curve analyses and predicted probability distributions for the binary Oswestry Disability Index model across (A and B) NORspine, (C and D) SweSpine, and (E and F) DaneSpine. In the predicted probability distributions, shaded areas represent logistic regression and dashed lines XGBoost predictions. PASS = Patient acceptable symptom state at 12 months; NON-PASS = Not achieving patient acceptable symptom state at 12 months.

Fig. 5 illustrates decision curve analysis and the predicted probability distributions for the binary ODI models in the three cohorts. The models showed consistent net benefit across a broad range of threshold probabilities for experiencing an acceptable symptom state in all cohorts. Both modelling approaches outperformed the reference strategy of treating all patients at threshold probabilities of 0.2 and above. Predicted probability distributions showed that patients experiencing an acceptable symptom state generally had higher predicted probabilities, whereas those not experiencing an acceptable symptom state were concentrated at lower predicted values. For the NRS pain models, decision curve analysis indicated generally lower net benefit and less distinct separation between predicted probability distributions compared with ODI, particularly for leg pain (Supplementary Figs. S8 and S9).

Model performance was consistent across subgroups defined by fusion surgery and sex (Table 3 and Supplementary Table S7). For both continuous and binary models, performance metrics were similar to those observed in the overall analyses, with no meaningful differences between males and females or within the subgroup of patients who underwent fusion surgery. Sensitivity analyses based on complete cases yielded results consistent with the main analyses (Supplementary Table S8).

**Table 3:** Performance metrics with 95% confidence intervals for statistical models stratified by surgical procedure and sex.

	Decomp. alone (n = 27,648)	Decomp. with fusion (n = 4260)	Men (n = 15,191)	Women (n = 16,717)
Continuous models				
ODI				
MAE	12.4 (12.1, 12.7)	13.2 (12.8, 13.5)	12.2 (11.9, 12.5)	12.7 (12.5, 13.0)
Adjusted R^2^	0.29 (0.28, 0.30)	0.26 (0.24, 0.27)	0.29 (0.28, 0.30)	0.27 (0.26, 0.28)
CITL	0.47 (−0.27, 1.22)	−1.34 (−2.35, −0.33)	0.15 (−0.59, 0.88)	0.31 (−0.36, 0.98)
C-slope	1.02 (0.98, 1.05)	0.99 (0.94, 1.05)	1.01 (0.96, 1.05)	1.00 (0.97, 1.03)
NRS back				
MAE	2.17 (2.15, 2.19)	2.13 (2.08, 2.18)	2.15 (2.12, 2.18)	2.18 (2.15, 2.21)
Adjusted R^2^	0.16 (0.15, 0.17)	0.13 (0.12, 0.14)	0.17 (0.16, 0.18)	0.13 (0.13, 0.14)
CITL	0.06 (−0.04, 0.17)	−0.28 (−0.39, −0.17)	0.00 (−0.10, 0.10)	0.04 (−0.06, 0.13)
C-slope	1.02 (0.98, 1.05)	0.94 (0.86, 1.03)	1.01 (0.96, 1.05)	0.97 (0.93, 1.01)
NRS leg				
MAE	2.36 (2.34, 2.39)	2.41 (2.36, 2.45)	2.30 (2.27, 2.34)	2.43 (2.40, 2.46)
Adjusted R^2^	0.12 (0.12, 0.13)	0.10 (0.09, 0.11)	0.13 (0.12, 0.14)	0.10 (0.09, 0.11)
CITL	0.07 (−0.04, 0.19)	−0.33 (−0.45, −0.20)	0.00 (−0.11, 0.11)	0.04 (−0.07, 0.15)
C-slope	1.02 (0.98, 1.07)	0.94 (0.85, 1.04)	1.02 (0.96, 1.08)	0.97 (0.92, 1.03)
Binary models				
ODI				
C-statistic	0.76 (0.76, 0.77)	0.75 (0.73, 0.76)	0.77 (0.76, 0.78)	0.75 (0.74, 0.76)
CITL	−0.06 (−0.14, 0.03)	0.18 (0.08, 0.29)	−0.01 (−0.10, 0.08)	−0.03 (−0.11, 0.05)
C-slope	1.01 (0.97, 1.04)	0.97 (0.89, 1.04)	1.00 (0.95, 1.04)	0.98 (0.94, 1.02)
NRS back				
C-statistic	0.70 (0.69, 0.71)	0.68 (0.66, 0.70)	0.71 (0.70, 0.71)	0.68 (0.67, 0.69)
CITL	−0.04 (−0.11, 0.04)	0.18 (0.09, 0.27)	0.00 (−0.07, 0.08)	−0.02 (−0.10, 0.05)
C-slope	1.01 (0.96, 1.05)	0.96 (0.85, 1.07)	1.01 (0.95, 1.07)	0.95 (0.89, 1.01)
NRS leg				
C-statistic	0.67 (0.67, 0.68)	0.66 (0.65, 0.68)	0.68 (0.68, 0.69)	0.66 (0.65, 0.67)
CITL	−0.04 (−0.11, 0.03)	0.21 (0.12, 0.29)	0.00 (−0.07, 0.08)	−0.02 (−0.09, 0.05)
C-slope	1.01 (0.95, 1.07)	0.92 (0.82, 1.04)	1.05 (0.97, 1.12)	0.92 (0.85, 0.99)

Decomp = Decompression; ODI = Oswestry Disability Index; NRS = Numeric Rating Scale; MAE = mean absolute error; CITL = calibration-in-the-large; C-slope = calibration slope.

## Discussion

We developed and externally validated statistical and machine learning models predicting postoperative disability and pain following surgery for lumbar spinal stenosis using large-scale data from three national spine registries. Across both continuous and binary outcome definitions, models predicting disability demonstrated good overall performance, explaining up to 34% of the outcome variance and achieving C-statistics up to 0.78 at external validation. The models generalised well both within Norway and in external validation cohorts from Sweden and Denmark.

Prediction modelling in spine surgery has advanced rapidly in recent years, yet most efforts remain at the development stage, with limited integration into clinical practice.^43^ Impact studies are needed to determine whether prediction models can improve decision-making and patient outcomes, but such evaluations should not be undertaken until the robustness and generalisability of a model have been demonstrated.^44^ This study shows that prediction models based on routinely collected registry data can perform consistently across populations, providing a foundation for future assessment of their clinical utility. In addition to stable performance, decision curve analyses demonstrated consistent net benefit across cohorts, suggesting that the models may provide clinical value across a broad range of threshold probabilities. However, these analyses represent an intermediate step towards understanding the potential impact on care processes and patient outcomes.^45^

Compared with previous prediction models developed for lumbar spinal stenosis surgery,^8^, ^9^, ^10^, ^11^, ^12^ the binary models showed comparable discrimination, with validated C-statistics of ≥0.75 for ODI across all three national registries. Modelling continuous outcomes has been uncommon in spine surgery and prediction modelling more generally, despite its advantages of preserving information that is lost through dichotomisation, allowing more precise estimation of expected postoperative status. In the present study, the prediction error of approximately 12 ODI points represents the typical deviation between predicted and observed disability scores. While 12 points on a 0–100 scale indicates some uncertainty at the individual level, the predictions remain clinically informative for communicating expected recovery and likely ranges of improvement. Importantly, the models are intended to support structured preoperative counselling by providing individualized predicted disability estimates, thereby facilitating shared decision-making and expectation management rather than determining surgical indication alone. Such information may help align treatment decisions with expected outcomes, for example by identifying patients with a poor prognosis who may choose to avoid surgery, and those with a more favourable prognosis who may be more likely to benefit. This may contribute to reducing ineffective and costly spine care while improving outcomes among patients undergoing surgery. The probability of achieving an acceptable symptom state may be included as complementary information based on the continuous probability estimate, which may be more informative than categorizing patients into risk strata in the context of counselling and shared decision-making.^46^ However, there is limited knowledge about how such predictions are perceived and used by patients and surgeons in routine clinical practice, and whether their use influences recommendations and decision-making. This is currently being explored in ongoing feasibility and pilot studies in Norway and Sweden.^21^^,^^47^ The ODI model further showed a small systematic underprediction of 1.3–1.5 points in the external data, indicating that predicted scores were slightly lower than observed scores. Differences between predicted and observed scores were also minor across Norwegian public health regions (CITL −0.7 to 1.5 points) and unlikely to be clinically meaningful considering the ODI scale from 0 to 100. However, slightly larger deviations were observed in private hospitals, underscoring the importance of validating models across care settings within the development data to ensure applicability in the intended clinical context. For pain outcomes, higher relative prediction error and lower discriminatory performance indicate greater uncertainty in individual predictions, which may limit standalone usefulness of the models, particularly for leg pain. This might reflect both the lower reliability of single-item pain measures compared with the multidimensional ODI and the greater inherent fluctuation in pain symptoms relative to disability.

Our models’ performance and consistent generalisability across cohorts contrasts with earlier models for lumbar spinal stenosis that have shown limited transportability outside their development setting, although our validation so far has been restricted to the Scandinavian context. Model performance was even slightly higher in the external cohorts, including an explained variance of 34% for ODI, which is considered high in clinical medicine.^48^ This can be explained by differences in case-mix and evaluation setting. Internal-external validation is intentionally conservative because the model must generalise across potentially heterogeneous regions, and the pooled performance estimate reflects this variation.^40^ In contrast, when models are evaluated in a large national cohort, the distributions of predictors and outcomes may show clearer separation between patients with low and high disability or pain levels, contributing to higher explained variance or discrimination. Other externally validated models, such as the SCOAP-CERTAIN^15^^,^^17^ and FUSE-ML^10^ demonstrated acceptable discrimination but poor calibration at external validation. These models were also developed for fusion procedures, which narrows their scope given the limited evidence supporting added benefit of fusion over decompression alone and the inability to readily apply such models to the larger population undergoing decompression without fusion.^49^ The present models performed consistently in subgroup analyses stratified by surgical procedure (decompression alone and decompression with fusion) and sex, supporting the robustness of predictions across different subgroups. Another externally validated model, the streamlined Quality Outcomes Database web-calculator,^50^ was developed for a broader range of spine procedures but showed limited ability to discriminate between patients achieving a 30% improvement in disability or pain in a distinct international cohort, with C-statistics ranging from 0.62 to 0.63.^16^

Both the machine learning (XGBoost) and traditional regression models produced nearly identical estimates of discrimination and calibration across outcomes and datasets, indicating that the underlying associations between the 16 predictors and postoperative outcomes were adequately captured by conventional regression models. This finding aligns with previous evidence showing that complex algorithms do not markedly outperform simpler, well-specified statistical models when applied to structured data.^51^ However, machine learning may offer advantages in settings involving more complex structured data and a greater number of candidate predictors.

This study has limitations. Although based on large, prospectively collected registry data, the registries do not capture all potentially relevant predictors. Residual confounding and unmeasured case-mix differences across countries may therefore have affected predictive performance. Furthermore, our predictor set was limited to variables harmonized across registries to enable external validation. Although this strengthens international applicability, a full model within one registry may achieve slightly higher performance. Limited granularity in factors relevant to surgical planning (e.g., detailed radiographic findings) may also partly explain the moderate predictive performance. We also deliberately restricted predictors to variables reliably available preoperatively to align the models with the intended decision-making context. Second, while missingness of predictors was low, missing outcome data ranged from 20% to 34% depending on outcome and cohort. We addressed missing data using multiple imputation under the missing-at-random assumption. Previous analyses from NORspine and DaneSpine have indicated limited differences in patient-reported outcomes between respondents and non-respondents at follow-up.^28^^,^^52^ Under the missing-at-random assumption and with a correctly specified imputation model, multiple imputation yields unbiased estimates.^35^ While analyses from SweSpine have suggested that non-respondents may have slightly worse outcomes,^53^ sensitivity analyses using a complete-case approach also yielded similar performance estimates. Third, despite rigorous internal-external cross-validation and external validation across three national registries, the models were validated only within the Scandinavian context, and their performance in other healthcare systems remains unknown. Accordingly, the models demonstrate the potential for data-driven tools to support shared decision-making and warrant further impact testing in this context, but application in other settings would require additional local validation. Future research should include impact studies assessing clinical utility and cost-effectiveness to inform implementation in routine care.^45^ Fourth, outcomes were assessed by patient-reported measures, which are inherently subjective but reflect the outcomes most relevant to patients. Other outcomes, such as the risk of complications, are also important considerations in the surgical decision-making process. Finally, the models are prognostic and not causal. They are intended to inform expectations and shared decision-making, rather than to dictate or replace clinical judgment, which on its own has shown modest accuracy in predicting individual postoperative outcomes.^5^

For clinical implementation, the models would require deployment within decision support tools integrated into electronic health records to provide individualized estimates at the point of preoperative consultation. Stand-alone tools that operate outside routine clinical workflows may limit adoption, as decision support is more likely to be used when embedded within existing systems with minimal interference with clinical practice.^54^ Such integration should be accompanied by prospective evaluation of usability and clinical impact prior to wider deployment. Application in other populations will require additional local validation and potentially recalibration, given the minor calibration drift observed at external validation within the Scandinavian context. Following implementation, periodic performance monitoring and model updating should be considered.

In summary, we developed and externally validated multivariable models predicting postoperative disability and pain following lumbar spinal stenosis surgery using large-scale registry data from three national spine registries. The disability models demonstrated the best performance, with consistent generalisability across healthcare settings and countries in Scandinavia. These findings support the use of individualized prognostic information to enhance shared decision-making and promote more informed and personalised surgical care.

## Contributors

Conceptualization: BB, TI, TS, MG. Data curation: BB, AA, CFP, HH, HLJ, PJB, KØM, MØA, TI, TS. Formal analysis: BB. Funding acquisition: MG. Investigation: BB. Methodology: BB. Project administration: MG. Resources: AA, CFP, HH, MØA, TI, TS. Supervision: TS, MG. Validation: MAG. Visualization: BB. Writing – original draft: BB. Writing – review & editing: AA, CFP, HH, MAG, HLJ, PJB, KØM, MØA, TI, TS, MG. BB and MG have accessed and verified the data, and were responsible for the decision to submit the manuscript. All authors read and approved the final manuscript.

## Data sharing statement

Individual-level patient data used in the analysis are not freely available due to institutional and regulatory requirements concerning patient privacy and data protection but may be obtained through application to the respective registry holders. Analysis code used to generate the results is available in repository https://github.com/bjornarb88/AID-Spine-LSS-models.

## Declaration of interests

HLJ reports collaboration with the electronic health record vendor DIPS ASA, without direct financial compensation. TI reports being a member of the Advisory Board for the Norwegian Spine Registry. MØA reports travel support from EUROSPINE for attending meetings. All other authors declare no competing interests.

## Appendix A Supplementary data

Supplementary Figs. S1–S11 and Tables S1–S8

Protocol
