# Supplementary material for: Generalisable prediction models for outcomes after lumbar spinal stenosis surgery: a model development and external validation study
Source: eClinicalMedicine. 2026 May 28;96:103989. doi: 10.1016/j.eclinm.2026.103989 (PMC13233580; doi:10.1016/j.eclinm.2026.103989)
Supplement: Supplementary Figs. S1–S11 and Tables S1–S8 [file mmc1.docx]

**Supplementary material**

**Extended methods** – Sample size calculation

**Supplementary Table S1.** Description of predictors

**Supplementary Table S2.** Number (%) of missing values in development and validation cohorts

**Supplementary Table S3.** Hyperparameters of machine learning models

**Supplementary Table S4.** Coefficients and constant term from linear regression models

**Supplementary Table S5.** Coefficients and constant term from logistic regression models

**Supplementary Table S6.** Performance metrics with 95% confidence intervals of XGBoost models

**Supplementary Table S7.** Performance metrics with 95% confidence intervals in patients undergoing fusion surgery and stratified by sex

**Supplementary Table S8.** Performance metrics with 95% confidence intervals in complete-case sensitivity analyses

**Supplementary Figure S1**. Illustration of internal-external cross-validation

**Supplementary Figure S2.** Internal-external cross-validation in five regional validation cohorts and the overall estimation across cohorts for Numeric Rating Scale back pain continuous model from (A) Linear regression and (B) XGBoost

**Supplementary Figure S3.** Internal-external cross-validation in five regional validation cohorts and the overall estimation across cohorts for Numeric Rating Scale leg pain continuous model from (A) Linear regression and (B) XGBoost

**Supplementary Figure S4.** Internal-external cross-validation in five regional validation cohorts and the overall estimation across cohorts for Numeric Rating Scale back pain binary patient acceptable symptom state models from (A) Linear regression and (B) XGBoost

**Supplementary Figure S5.** Internal-external cross-validation in five regional validation cohorts and the overall estimation across cohorts for Numeric Rating Scale leg pain binary patient acceptable symptom state models from (A) Linear regression and (B) XGBoost

**Supplementary Figure S6.** Model calibration for linear regression models across five regional validation cohorts from internal-external cross-validation (A) Oswestry Disability Index, (B) Numeric Rating Scale back pain, and (C) Numeric Rating Scale leg pain

**Supplementary Figure S7.** Model calibration for logistic regression models across five regional validation cohorts from internal-external cross-validation (A) Oswestry Disability Index, (B) Numeric Rating Scale back pain, and (C) Numeric Rating Scale leg pain

**Supplementary Figure S8.** Model calibration in external validation data for NRS back pain, with continuous models in (A) SweSpine and (B) DaneSpine, and binary models in (C) SweSpine and (D) DaneSpine

**Supplementary Figure S9.** Model calibration in external validation data for NRS leg pain, with continuous models in (A) SweSpine and (B) DaneSpine, and binary models in (C) SweSpine and (D) DaneSpine

**Supplementary Figure S10.** Decision curve analyses and predicted probability distributions for the binary NRS back pain model across (A) NORspine, (B) SweSpine, and (C) DaneSpine. In the predicted probability distributions, shaded areas represent logistic regression and dashed lines XGBoost predictions.

**Supplementary Figure S11.** Decision curve analyses and predicted probability distributions for the binary NRS leg pain model across (A) NORspine, (B) SweSpine, and (C) DaneSpine. In the predicted probability distributions, shaded areas represent logistic regression and dashed lines XGBoost predictions.

**Extended methods** – Sample size calculation

Sample size calculations followed the framework proposed by Riley et al. for prediction model development^1^ and external validation.^2^ While the sample size in our cohorts were fixed, the calculations aimed to ensure sufficiently precise estimates of model performance measures and limit overfitting.

For model development, our prespecified sample size calculation was based a maximum number of 44 predictor parameters (to allow for all predictors plus possible transformation of all continuous variables). Assuming an R^2^ of 0.30, a mean ODI score of 23.3, and a standard deviation of 18.2, a sample size of 1,052 surgical cases was estimated to be required for the continuous ODI model. For binary models, we estimated at least 2,078 cases (with 977 events) would be required, based on an outcome prevalence of 47% and a C-statistic of 0.74.^3^ In our analysis, we in fact considered a total of 33 predictor parameters, which instead required a sample size of 775 cases for the continuous ODI model and 1,558 (with 733 events) for binary outcomes. Our development sample size in NORspine far exceeded this.

For external validation, input values from the developed models were used to confirm sufficient power for accurate discrimination and calibration assessment.^2^ For the continuous ODI model, with an R^2^ of 0.28, conservatively assumed perfect calibration, and an outcome standard deviation of 18.2, a minimum of 990 cases was required to achieve confidence interval (CI) widths of 0.1 for R^2^, 5 for calibration-in-the-large (considered precise given the ODI score scale), and 0.2 for the calibration slope. For binary outcomes, with a normally distributed linear predictor (mean=0.18, SD=1.13), a prevalence of 47%, and a C-statistic of 0.76, at least 2,340 cases (1,100 events) were required to achieve default CI widths. The sample sizes in both external validation cohorts exceeded this.

**Supplementary Table S1.** Description of predictors

| **Variables (functional form)** | **Description** | **Cohort differences** |
| --- | --- | --- |
| Sex (binary) | Male or female |  |
| Age (continuous) | In years |  |
| Body mass index (continuous) | Self-reported height and weight, in kg/m^2^ |  |
| Smoker (binary) | Smoker or non-smoker |  |
| Work Status (categorical) | Working/Student, Retirement, Sick leave, Welfare benefits |  |
| Back pain duration (categorical) | Self-reported duration of neck pain: None or less than 3 months, 3 to 11 months, 12 to 24 months, More than 24 months. |  |
| Leg pain duration (categorical) | Self-reported duration of arm pain: None or less than 3 months, 3 to 11 months, 12 to 24 months, More than 24 months. |  |
| ODI (continuous) | Oswestry Disability Index score (range 0- 100) |  |
| NRS back pain (continuous) | NRS for back pain (range 0 to 10) | DaneSpine: VAS (0-100) converted to NRS by dividing by 10 and rounding |
| NRS leg pain (continuous) | NRS for leg pain (range 0 to 10) | DaneSpine: VAS (0-100) converted to NRS by dividing by 10 and rounding |
| EQ-5D (continuous) | Health-related quality of life (5L). For patients responding to the 3L version, reverse crosswalk values were computed using the EQ-5D-5L Delvin value set (range -0.285 to 1) |  |
| EQ-VAS (continuous) | Self-reported health status using Visual Analogue Scale from EQ-5D (range 0 to 100) |  |
| Anxiety/Depression (binary) | Self-reported anxiety or depression, based on EQ-5D 5^th^ item: moderate to extreme |  |
| Previous surgery (categorical) | None, One, Two or more |  |
| Comorbidities (categorical) | Recorded by the surgeon, from a list of relevant comorbidities. Categorized as none, one, two, three or more | NORspine: more extensive list of comorbidities |
| Analgesics use (Categorical) | Self-reported frequency due to back pain: Monthly or less frequent, Weekly, Daily. | SweSpine: Not using, Occasionally, Regularly. DaneSpine: No, Less frequent, On a regular basis |

ODI=Oswestry Disability Index; NRS=Numeric Rating Scale.

**Supplementary Table S2.** Number (%) of missing values in development and validation cohorts

|  | **NORspine** (development)  (n=31,908) | **SweSpine** (validation) (n=30,700) | **DaneSpine** (validation) (n=4,063) |
| --- | --- | --- | --- |
| Sex | 0 | 0 | 0 |
| Age (years) | 46 (0.1) | 16 (0.1) | 0 |
| Body mass index | 1,241 (3.9) | 1,951 (6.4) | 12 (0.3) |
| Smoker | 316 (1.0) | 357 (1.2) | 91 (2.2) |
| Work status | 374 (1.2) | 79 (0.3) | 0 |
| Back pain | 2,355 (7.4) | 358 (1.2) | 84 (2.1) |
| Leg pain | 2,727 (8.6) | 265 (0.9) | 78 (1.9) |
| ODI | 537 (1.7) | 1,056 (3.4) | 132 (3.3) |
| NRS back pain | 1,409 (4.4) | 940 (3.1) | 119 (2.9) |
| NRS leg pain | 1,958 (6.1) | 857 (2.8) | 2 (0.1) |
| EQ5D | 2,021 (6.3) | 496 (1.6) | 153 (3.8) |
| EQ-VAS | 2,577 (8.1) | 2,830 (9.2) | 179 (4.4) |
| Anxiety/depression | 1,047 (3.3) | 494 (1.6) | 87 (2.1) |
| Previous surgery | 190 (0.6) | 241 (0.8) | 75 (1.9) |
| Comorbidities | 0 | 623 (2.0) | 106 (2.6) |
| Analgesics frequency | 577 (1.8) | 340 (1.1) | 640 (15.8) |
| ODI 12 months | 7,294 (22.9) | 10,492 (34.2) | 992 (24.4) |
| NRS back 12 months | 7,328 (23.0) | 10,219 (33.3) | 834 (20.5) |
| NRS leg 12 moths | 7,550 (23.7) | 10,417 (33.9) | 819 (20.2) |

ODI=Oswestry Disability Index; NRS=Numeric Rating Scale

**Supplementary Table S3.** Coefficients and constant term from linear regression models

| **Parameter** | **Description** | **Coefficient** | | |
| --- | --- | --- | --- | --- |
|  |  | **ODI** | **NRS back** | **NRS leg** |
| Sex | Male (ref) | 0 | 0 | 0 |
|  | Female | -0.186657 | 0.039544 | -0.029257 |
| Age | 1^st^ FP term | -0.124251 | -0.018199 | -0.010441 |
|  | 2^nd^ FP term | 0.058831 | 0.008033 | 0.004998 |
| BMI | Continuous (linear) | 0.217463 | 0.027881 | 0.021070 |
| Smoker | No (ref) | 0 | 0 | 0 |
|  | Yes | 3.379780 | 0.457942 | 0.405175 |
| Work status | Working/student (ref) | 0 | 0 | 0 |
|  | Age retirement | 1.342027 | 0.311156 | 0.216078 |
|  | Sick leave | 1.241698 | 0.249430 | 0.250555 |
|  | Welfare benefits | 4.549809 | 0.593914 | 0.592449 |
| Anxiety/depression | No (ref) | 0 | 0 | 0 |
|  | Yes | 2.979600 | 0.381165 | 0.494582 |
| Comorbidity | None (ref) | 0 | 0 | 0 |
|  | One | 0.507814 | 0.088612 | 0.114495 |
|  | Two | 1.457005 | 0.181391 | 0.150542 |
|  | Three or more | 2.366529 | 0.270636 | 0.327617 |
| ODI | Continuous (linear) | - | 0.013721 | 0.015711 |
|  | 1^st^ FP term | 93.480589 | - | - |
|  | 2^nd^ FP term | -64.817817 | - | - |
| NRS back pain | Continuous (linear) | 0.373206 | 0.236593 | 0.043032 |
| NRS leg pain | Continuous (linear) | -0.476410 | -0.040082 | 0.168684 |
| EQ5D | Continuous (linear) | -2.693184 | -0.391833 | 0.048662 |
| EQ-VAS | 1^st^ FP term | -2.879786 | -0.428916 | 1.344287 |
|  | 2 ^st^ FP term | - | -1.334283 | -1.531846 |
| Back pain duration | < 3 months (ref) | 0 | 0 | 0 |
|  | 3 to 12 months | 0.211924 | 0.089030 | -0.055688 |
|  | 13 to 24 months | 2.822312 | 0.478003 | 0.226016 |
|  | > 24 months | 4.878606 | 0.831721 | 0.414122 |
| Leg pain duration | < 3 months (ref) | 0 | 0 | 0 |
|  | 3 to 12 months | 0.786943 | 0.065777 | 0.027711 |
|  | 13 to 24 months | 2.301943 | 0.186192 | 0.316687 |
|  | > 24 months | 3.548401 | 0.312291 | 0.587877 |
| Analgesics use | Monthly (ref) | 0 | 0 | 0 |
|  | Weekly | 0.829285 | 0.235900 | 0.151123 |
|  | Daily | 1.552301 | 0.322216 | 0.197584 |
| Previous surgery | Zero (ref) | 0 | 0 | 0 |
|  | One | 4.984829 | 0.654852 | 0.665846 |
|  | Two or more | 6.744798 | 0.865294 | 0.929391 |
| Constant |  | 6.941664 | -0.330768 | -0.232589 |

Fractional Polynomial terms:
ODI model: Age FP1 = (X^3)-276.17, Age FP2 = (X^*3)(ln(X))-517.45, X=age/10;

ODI FP1 = (X^-2)-0.1513, ODI FP2 = (X^3)-0.0599, X=(odi+1)/100

EQ-VAS FP1 = (X^3)-0.1282, X=(EQ-VAS+1)/100

NRS back model: Age FP1 = (X^3)-276.27, Age FP2 = (X^3)*(ln(X))-517.67, X=age/10;

EQ-VAS FP1 = (X^3)-0.1282, EQVAS FP2 = (X^3)*ln(X))+0.0878, X=(EQVAS+1)/100

NRS leg model: Age FP1 = (X^3)-275.74, Age FP2 = (X^3)*(ln(X))-516.50, X=age/10

EQ-VAS FP1 = (X^2)-0.2546, EQVAS FP2 = (X^3)*ln(X))+0.1284, X=(EQVAS+1)/100

**Supplementary Table S4.** Hyperparameters of machine learning models

| **XGBoost, reg:squarederror** | **Range explored** | **Selected value** | | |
| --- | --- | --- | --- | --- |
|  |  | **ODI** | **NRS back** | **NRS leg** |
| Max depth | 1, 2, 3, 4 | 3 | 4 | 2 |
| Learning rate (eta) | 0.01-0.1 | 0.072 | 0.01 | 0.01 |
| No of boosting rounds | 1-2000 | 228 | 932 | 1956 |
| Reg alpha | 0-3 | 1.259 | 3 | 0.571 |
| Reg lambda | 0-5 | 0.760 | 0.031 | 3.204 |
| Reg gamma | 0-6 | 2.018 | 5.927 | 2.251 |
| Colum sampling by tree | 0.4-0.8 | 0.665 | 0.431 | 0.4 |
| **XGBoost, binary:logistic** |  |  |  |  |
| Max depth | 1, 2, 3, 4 | 3 | 2 | 1 |
| Learning rate (eta) | 0.01-0.1 | 0.033 | 0.039 | 0.096 |
| No of boosting rounds | 1-2000 | 473 | 497 | 197 |
| Reg alpha | 0-3 | 0.336 | 2.491 | 2.936 |
| Reg lambda | 0-5 | 3.192 | 1.155 | 5 |
| Reg gamma | 0-6 | 1.366 | 0.247 | 1.311 |
| Colum sampling by tree | 0.4-0.8 | 0.585 | 0.406 | 0.770 |

ODI=Oswestry Disability Index, NRS=Numeric Rating Scale

**Supplementary Table S5.** Coefficients and constant term from logistic regression models

| **Parameter** | **Description** | **Coefficient** | | |
| --- | --- | --- | --- | --- |
|  |  | **ODI** | **NRS back** | **NRS leg** |
| Sex | Male (ref) | 0 | 0 | 0 |
|  | Female | 0.001661 | -0.048750 | 0.028326 |
| Age | 1^st^ FP term | 0.014288 | 0.012430 | 0.005987 |
|  | 2^nd^ FP term | -0.006897 | -0.005589 | -0.002985 |
| BMI | Continuous (linear) | -0.028696 | -0.022148 | -0.015583 |
| Smoker | No (ref) | 0 | 0 | 0 |
|  | Yes | -0.416472 | -0.359261 | -0.281495 |
| Work status | Working/student (ref) | 0 | 0 | 0 |
|  | Age retirement | -0.256543 | -0.251527 | -0.168528 |
|  | Sick leave | -0.210387 | -0.221430 | -0.199453 |
|  | Welfare benefits | -0.578513 | -0.477374 | -0.390936 |
| Anxiety/depression | No (ref) | 0 | 0 | 0 |
|  | Yes | -0.337834 | -0.282530 | -0.314093 |
| Comorbidity | None (ref) | 0 | 0 | 0 |
|  | One | -0.057703 | -0.063640 | -0.052809 |
|  | Two | -0.160066 | -0.134187 | -0.089457 |
|  | Three or more | -0.237529 | -0.210610 | -0.190734 |
| ODI | Continuous (linear) | - | -0.007162 | -0.008701 |
|  | 1^st^ FP term | -6.324228 | - |  |
|  | 2^nd^ FP term | 1.625637 | - | - |
| NRS back pain | Continuous (linear) | -0.051134 | - | -0.033766 |
|  | Continuous (linear) | - | -1.605253 | - |
|  | Continuous (linear) | - | 2.499666 | - |
| NRS leg pain | 1^st^ FP term | 0.054468 | 0.018820 | -0.103930 |
| EQ5D | 2 ^st^ FP term | 0.202852 | 0.252541 | -0.026057 |
| EQ-VAS | < 3 months (ref) | 0.435278 | 0.219190 | -1.540635 |
|  | 3 to 12 months | - | 1.162378 | 1.764476 |
| Back pain duration | 13 to 24 months | 0 | 0 | 0 |
|  | > 24 months | -0.010662 | -0.114077 | 0.063392 |
|  | < 3 months (ref) | -0.319683 | -0.407531 | -0.158691 |
|  | 3 to 12 months | -0.558524 | -0.646302 | -0.280153 |
| Leg pain duration | 13 to 24 months | 0 | 0 | 0 |
|  | > 24 months | -0.093306 | -0.048753 | -0.058372 |
|  | Monthly (ref) | -0.264602 | -0.120432 | -0.231392 |
|  | Weekly | -0.409431 | -0.207033 | -0.391602 |
| Analgesics use | Daily | 0 | 0 | 0 |
|  | Zero (ref) | -0.139950 | -0.167980 | -0.121131 |
|  | One | -0.205364 | -0.240724 | -0.161370 |
| Previous surgery | Two or more | 0 | 0 | 0 |
|  |  | -0.599531 | -0.469733 | -0.451407 |
|  | Male (ref) | -0.781710 | -0.606701 | -0.614314 |
| Constant | Female | 2.271679 | 3.413238 | 2.441582 |

Fractional Polynomial terms:
ODI model: Age FP1 = (X^3)-276.17, Age FP2 = (X^3)*(ln(X))-517.45, X=age/10;

ODI FP1 = (X^0.5)-0.6237, ODI FP2 = (X^3)-0.0589, X=(ODI+0.1)/100;
 EQ-VAS FP1 = (X^3)-0.1282, X=(EQ-VAS+1)/100

NRS back model: Age FP1 = (X^3)-276.27, Age FP2 = (X^3)*(ln(X))-517.67, X=age/10;
NRS back FP1 = (X^2)-0.5690; NRS back FP2 = (X^2)*ln(X)-0.4292, X=(NRS back+1)/10);

EQ-VAS FP1 = (X^2)-0.2543, EQVAS FP2 = (X^2)*ln(X)+0.1741, X=(EQVAS+1)/100

NRS leg model: Age FP1 = (X^3)-275.74, Age FP2 = (X^3)*(ln(X))-516.50, X=age/10;

EQ-VAS FP1 = (X^2)-0.2546, EQVAS FP2 = (X^3)+0.1284, X=(EQVAS+1)/100

**Supplementary Table S6.** Performance metrics with 95% confidence intervals for XGBoost models

|  | **NORspine (n=31,908)^a^** | **SweSpine (n=30,700)** | **DaneSpine (n=4,063)** |
| --- | --- | --- | --- |
| **Continuous models** |  |  |  |
| ODI |  |  |  |
| MAE | 12.4 (11.8, 13.0) | 13.2 (13.1, 13.4) | 12.3 (12.0, 12.6) |
| Adjusted R^2^ | 0.26 (0.23, 0.29) | 0.34 (0.33, 0.35) | 0.28 (0.26, 0.30) |
| CITL | -0.50 (-2.62, 1.62) | 1.43 (1.21, 1.65) | 1.33 (0.80, 1.86) |
| C-slope | 0.97 (0.91, 1.04) | 1.17 (1.15, 1.19) | 0.97 (0.92, 1.03) |
| NRS back pain |  |  |  |
| MAE | 2.17 (2.15, 2.19) | 2.22 (2.20, 2.24) | 2.29 (2.24, 2.34) |
| Adjusted R^2^ | 0.14 (0.11, 0.16) | 0.21 (0.20, 0.22) | 0.19 (0.18, 0.21) |
| CITL | -0.05 (-0.34, 0.25) | -0.18 (-0.21, -0.14) | -0.32 (-0.41, -0.23) |
| C-slope | 0.99 (0.91, 1.06) | 1.17 (1.14, 1.20) | 1.12 (1.04, 1.20) |
| NRS leg pain |  |  |  |
| MAE | 2.36 (2.30, 2.43) | 2.51 (2.49, 2.53) | 2.60 (2.55, 2.55) |
| Adjusted R^2^ | 0.10 (0.07, 0.12) | 0.14 (0.13, 0.14) | 0.08 (0.06, 0.09) |
| CITL | -0.04 (-0.34, 0.25) | 0.02 (-0.02, 0.06) | 0.14 (0.04, 0.25) |
| C-slope | 0.97 (0.87, 1.07) | 1.17 (1.14, 1.21) | 1.03 (0.91, 1.16) |
| **Binary models** |  |  |  |
| ODI |  |  |  |
| C-statistic | 0.75 (0.74, 0.76) | 0.78 (0.78, 0.79) | 0.76 (0.74, 0.77) |
| CITL | 0.05 (-0.23, 0.34) | -0.07 (-0.10, -0.04) | -0.11 (-0.19, -0.03 |
| C-slope | 0.99 (0.95, 1.03) | 1.20 (1.16, 1.23) | 1.02 (0.93, 1.11) |
| NRS back pain |  |  |  |
| C-statistic | 0.69 (0.67, 0.70) | 0.73 (0.72, 0.73) | 0.73 (0.71, 0.75) |
| CITL | 0.03 (-0.21, 0.26) | 0.19 (0.16, 0.22) | 0.22 (0.15, 0.30) |
| C-slope | 0.99 (0.93, 1.05) | 1.19 (1.15, 1.24) | 1.15 (1.04, 1.26) |
| NRS leg pain |  |  |  |
| C-statistic | 0.66 (0.65, 0.68) | 0.68 (0.68, 0.69) | 0.64 (0.62, 0.66) |
| CITL | 0.03 (-0.19, 0.24) | 0.08 (0.05, 0.11) | -0.04 (-0.11, 0.04) |
| C- slope | 0.97 (0.90, 1.03) | 1.27 (1.21, 1.32) | 1.00 (0.85, 1.16) |

^a^Internal-external cross-validation estimates pooled across regions using random-effects meta-analysis
ODI=Oswestry Disability Index; NRS=Numeric Rating Scale; MAE=mean absolute error; CITL=calibration-in-the-large; C-slope=calibration slope

**Supplementary Table S7.** Performance metrics with 95% confidence intervals for XGBoost models stratified by surgical approach and sex

|  | **Decomp. alone (n=27,648)** | **Decomp. with fusion (n=4,260)** | **Men  (n=15,191)** | **Women  (n=16,717)** |
| --- | --- | --- | --- | --- |
| **Continuous models** |  |  |  |  |
| **ODI** |  |  |  |  |
| MAE | 12.4 (12.3, 12.5) | 13.1 (12.8, 13.5) | 12.2 (12.0, 12.4) | 12.7 (12.6, 12.9) |
| Adj R^2^ | 0.29 (0.28, 0.30) | 0.26 (0.24, 0.28) | 0.30 (0.29, 0.31) | 0.27 (0.26, 0.28) |
| CITL | 0.45 (0.24, 0.66) | -1.32 (-1.87, -0.78) | 0.21 (-0.07, 0.48) | 0.22 (-0.06, 0.50) |
| C-slope | 1.03 (1.01, 1.05) | 0.97 (0.91, 1.02) | 1.03 (1.00, 1.06) | 1.00 (0.97, 1.03) |
| **NRS back** |  |  |  |  |
| MAE | 2.18 (2.16, 2.20) | 2.14 (2.09, 2.18) | 2.16 (2.13, 2.18) | 2.19 (2.16, 2.21) |
| Adj R^2^ | 0.16 (0.15, 0.17) | 0.13 (0.12, 0.14) | 0.17 (0.16, 0.17) | 0.13 (0.13, 0.14) |
| CITL | 0.06 (0.03, 0.10) | -0.26 (-0.34, -0.17) | 0.00 (-0.05, 0.04) | 0.04 (-0.01, 0.08) |
| C-slope | 1.06 (1.03, 1.09) | 0.97 (0.88, 1.05) | 1.05 (1.01, 1.09) | 1.01 (0.97, 1.06) |
| **NRS leg** |  |  |  |  |
| MAE | 2.37 (2.35, 2.39) | 2.41 (2.36, 2.45) | 2.31 (2.28, 2.33) | 2.34 (2.41, 2.46) |
| Adj R^2^ | 0.12 (0.12, 0.13) | 0.10 (0.09, 0.11) | 0.13 (0.12, 0.14) | 0.10 (0.09, 0.11) |
| CITL | 0.07 (0.04, 0.11) | -0,32 (-0.42, -0.23) | 0.01 (-0.04, 0.07) | 0.02 (-0.03, 0.07) |
| C-slope | 1.06 (1.03, 1.10) | 0.95 (0.86, 1.04) | 1.07 (1.02, 1.11) | 1.00 (0.95, 1.05) |
| **Binary models** |  |  |  |  |
| **ODI** |  |  |  |  |
| C-statistic | 0.76 (0.75, 0.77) | 0.75 (0.73, 0.76) | 0.77 (0.76, 0.77) | 0.75 (0.74, 0.75) |
| CITL | -0.05 (-0.08, -0.02) | 0.17 (0.10, 0.24) | -0.02 (-0.06, 0.02) | -0.03 (-0.07, 0.01) |
| C-slope | 1.03 (0.99, 1.06) | 0.98 (0.90, 1.07) | 1.02 (0.98, 1.06) | 1.00 (0.96, 1.05) |
| **NRS back** |  |  |  |  |
| C-statistic | 0.70 (0.69, 0.70) | 0.68 (0.66, 0.70) | 0.70 (0.69, 0.71) | 0.68 (0.67, 0.69) |
| CITL | -0.03 (-0.06, 0.01) | 0.17 (0.10, 0.24) | 0.01 (-0.03, 0.05) | -0.03 (-0.06, 0.01) |
| C-slope | 1.06 (1.01, 1.10) | 1.01 (0.89, 1.12) | 1.05 (1.00, 1.11) | 1.01 (0.95, 1.07) |
| **NRS leg** |  |  |  |  |
| C-statistic | 0.67 (0.67, 0.68) | 0.66 (0.65, 0.68) | 0.68 (0.67, 0.69) | 0.66 (0.65, 0.67) |
| CITL | -0.04 (-0.07, -0.01) | 0.20 (0.13, 0.27) | -0.01 (-0.05, 0.03) | -0.01 (-0.04, 0.03) |
| C-slope | 1.07 (1.02, 1.12) | 0.99 (0.86, 1.11) | 1.09 (1.03, 1.16) | 0.99 (0.92, 1.05) |

Decom=Decompression; ODI=Oswestry Disability Index; NRS=Numeric Rating Scale; MAE=mean absolute error; CITL=calibration-in-the-large; C-slope=calibration slope

**Supplementary Table S8.** Performance metrics with 95% confidence intervals in complete-case sensitivity analyses

|  | **NORspine (IECV)*** | | **SweSpine** | | **DaneSpine** | |
| --- | --- | --- | --- | --- | --- | --- |
|  | **Statistical model** | **XGBoost** | **Statistical model** | **XGBoost** | **Statistical model** | **XGBoost** |
| **Cont. models** |  |  |  |  |  |  |
| **ODI** | **n=17,864** | **n=17,864** | **n=15,616** | **n=15,616** | **n=2,363** | **n=2,363** |
| MAE | 12.1 (11.4, 12.7) | 12.1 (11.4, 12.7) | 13.0 (12.8, 13.1) | 12.9 (12.8, 13.1) | 12.0 (11.7, 12.4) | 11.9 (11.5, 12.3) |
| Adjusted R^2^ | 0.26 (0.23, 0.30) | 0.27 (0.23, 0.31) | 0.33 (0.32, 0.35) | 0.35 (0.33, 0.36) | 0.26 (0.23, 0.29) | 0.27 (0.24, 0.30) |
| CITL | -0.48 (-2.38, 1.43) | -0.47 (-2.46, 1.52) | 1.27 (1.02, 1.52) | 1.73 (1.48, 1.98) | 1.57 (0.97, 2.17) | 1.30 (0.70, 1.90) |
| C-slope | 0.96 (0.86, 1.06) | 0.97 (0.91, 1.03) | 1.16 (1.14, 1.20) | 1.22 (1.19, 1.24) | 0.92 (0.86, 0.99) | 0.95 (0.89. 1.01) |
| **NRS back** | **n=17,856** | **n=17,856** | **n=15,791** | **n=15,791** | **n=2,452** | **n=2,452** |
| MAE | 2.14 (2.11, 2.16) | 2.15 (2.13, 2.17) | 2.17 (2.15, 2.20) | 2.19 (2.17, 2.21) | 2.22 (2.17, 2.28) | 2.26 (2.21, 2.32) |
| Adjusted R^2^ | 0.14 (0.11, 0.16) | 0.13 (0.11, 0.16) | 0.20 (0.19, 0.21) | 0.21 (0.19, 0.22) | 0.18 (0.15, 0.21) | 0.18 (0.15, 0.21) |
| CITL | -0.04 (-0.31, 0.23) | -0.05 (-0.34, 0.25) | -0.13 (0.17, -0.09) | -0.21 (-0.25, -0.17) | -0.30 (-0.40, -0.20) | -0.37 (-0.48, -0.27) |
| C-slope | 0.95 (0.91, 0.99) | 0.99 (0.91, 1.06) | 1.15 (1.11, 1.19) | 1.20 (1.17, 1.24) | 1.01 (0.93, 1.10) | 1.13 (1.04, 1.23) |
| **NRS leg** | **n=17,739** | **n=17,739** | **n=15,634** | **n=15,634** | **n=2,461** | **n=2,461** |
| MAE | 2.32 (2.24, 2.40) | 2.32 (2.26, 2.39) | 2.49 (2.46, 2.51) | 2.48 (2.46, 2.50) | 2.54 (2.47, 2.60) | 2.55 (2.49, 2.61) |
| Adjusted R^2^ | 0.11 (0.08, 0.14) | 0.10 (0.06, 0.13) | 0.13 (0.12, 0.14) | 0.14 (0.13, 0.15) | 0.06 (0.04, 0.08) | 0.06 (0.04, 0.08) |
| CITL | -0.04 (-0.31, 0.23) | -0.04 (-0.34, 0.25) | 0.01 (-0.03, 0.06) | 0.03 (-0.01, 0.08) | -0.03 (-0.14, 0.08) | 0.09 (-0.03, 0.20) |
| C-slope | 0.94 (0.83, 1.04) | 0.97 (0.87, 1.07) | 1.14 (1.10, 1.19) | 1.16 (1.11, 1.20) | 0.91 (0.78, 1.04) | 0.96 (0.83, 1.10) |
| **Binary models** |  |  |  |  |  |  |
| **ODI** | **n=17,864** | **n=17,864** | **n=15,616** | **n=15,616** | **n=2,363** | **n=2,363** |
| C-statistic | 0.76 (0.74, 0.77) | 0.76 (0.74, 0.77) | 0.78 (0.78, 0.79) | 0.78 (0.78, 0.79) | 0.75 (0.73, 0.77) | 0.75 (0.73, 0.77) |
| CITL | 0.05 (-0.20, 0.29) | 0.05 (-0.21, 0.31) | -0.07 (-0.11, -0.03) | -0.09 (-0.12, -0.05) | -0.17 (-0.25, -0.07) | -0.15 (-0.24, -0.06) |
| C-slope | 0.95 (0.88, 1.02) | 0.98 (0.88, 1.08) | 1.14 (1.10, 1.18) | 1.16 (1.12, 1.20) | 0.96 (0.86, 1.06) | 0.99 (0.89, 1.10) |
| **NRS back** | **n=17,856** | **n=17,856** | **n=15,791** | **n=15,791** | **n=2,452** | **n=2,452** |
| C-statistic | 0.69 (0.68, 0.71) | 0.69 (0.68, 0.70) | 0.72 (0.72, 0.73) | 0.72 (0.72, 0.73) | 0.73 (0.71, 0.75) | 0.72 (0.70, 0.74) |
| CITL | 0.02 (-0.19, 0.24) | 0.02 (-0.21, 0.25) | 0.14 (0.10, 0.17) | 0.21 (0.17, 0.24) | 0.19 (0.11, 0.28) | 0.24 (0.16, 0.33) |
| C-slope | 0.96 (0.91, 1.01) | 1.01 (0.94, 1.07) | 1.14 (1.09, 1.19) | 1.19 (1.13, 1.24) | 1.04 (0.92, 1.16) | 1.16 (1.03, 1.29) |
| **NRS leg** | **n=17,739** | **n=17,739** | **n=15,634** | **n=15,634** | **n=2,461** | **n=2,461** |
| C-statistic | 0.67 (0.65, 0.69) | 0.67 (0.64, 0.69) | 0.68 (0.67, 0.69) | 0.68 (0.67, 0.69) | 0.63 (0.61, 0.66) | 0.63 (0.61, 0.66) |
| CITL | 0.03 (-0.16, 0.22) | 0.03 (-0.18, 0.24) | 0.07 (0.04, 0.11) | 0.06 (0.03, 0.10) | -0.11 (-0.19, -0.03) | -0.09 (-0.17, -0.01) |
| C-slope | 0.92 (0.77, 1.07) | 0.97 (0.79, 1.15) | 1.05 (1.00, 1.11) | 1.04 (0.99, 1.10) | 0.81 (0.67, 0.95) | 0.88 (0.73, 1.04) |

Cont.=Continuous; ODI=Oswestry Disability Index; NRS=Numeric Rating Scale; CITL=calibration-in-the-large
*Pooled estimates from internal-external cross-validation obtained using random-effects meta-analysis with restricted maximum likelihood and Hartung-Knapp-Sidik-Jonkman adjustment.

**Supplementary Figure S1**. Illustration of internal-external cross-validation (adapted from Debray et al^4^). In each iteration, one region is held out for validation while the model is developed using data from the remaining regions. This process is repeated such that each region serve once as the validation set. Performance metrics are calculated separately for each held-out region and subsequently pooled using random-effects meta-analysis to obtain overall performance estimates.

**Supplementary Figure S2.** Internal-external cross-validation in five regional validation cohorts and the overall estimation across cohorts for Numeric Rating Scale back pain continuous model from (A) Linear regression and (B) XGBoost

 **Supplementary Figure S3.** Internal-external cross-validation in five regional validation cohorts and the overall estimation across cohorts for Numeric Rating Scale leg pain continuous model from (A) Linear regression and (B) XGBoost

 **Supplementary Figure S4.** Internal-external cross-validation in five regional validation cohorts and the overall estimation across cohorts for Numeric Rating Scale back pain binary patient acceptable symptom state model from (A) Logistic regression and (B) XGBoost

 **Supplementary Figure S5.** Internal-external cross-validation in five validation cohorts and the overall estimation across validation cohorts for Numeric Rating Scale leg pain binary patient acceptable symptom state model from (A) Logistic regression and (B) XGBoost


**Supplementary Figure S6.** Model calibration for linear regression models across five regional validation cohorts from internal-external cross-validation (A) Oswestry Disability Index, (B) Numeric Rating Scale back pain, and (C) Numeric Rating Scale leg pain

**Supplementary Figure S7.** Model calibration for logistic regression models across five regional validation cohorts from internal-external cross-validation (A) Oswestry Disability Index, (B) Numeric Rating Scale back pain, and (C) Numeric Rating Scale leg pain

** Supplementary Figure S8.** Model calibration in external validation data for NRS back pain, with continuous models in (A) SweSpine and (B) DaneSpine, and binary models in (C) SweSpine and (D) DaneSpine

** Supplementary Figure S9.** Model calibration in external validation data for NRS leg pain, with continuous models in (A) SweSpine and (B) DaneSpine, and binary models in (C) SweSpine and (D) DaneSpine

**
Supplementary Figure S10.** Decision curve analyses and predicted probability distributions for the binary NRS back pain model across (A-B) NORspine, (C-D) SweSpine, and (E-F) DaneSpine. In the predicted probability distributions, shaded areas represent logistic regression and dashed lines XGBoost predictions.

** Supplementary Figure S11.** Decision curve analyses and predicted probability distributions for the binary NRS leg pain model across (A-B) NORspine, (C-D) SweSpine, and (E-F) DaneSpine. In the predicted probability distributions, shaded areas represent logistic regression and dashed lines XGBoost predictions.

**References**

1. Riley RD, Ensor J, Snell KIE, et al. Calculating the sample size required for developing a clinical prediction model. *BMJ* 2020; **368**: m441.

2. Riley RD, Snell KIE, Archer L, et al. Evaluation of clinical prediction models (part 3): calculating the sample size required for an external validation study. *BMJ* 2024; **384**: e074821.

3. Geere JH, Hunter PR, Swamy GN, Cook AJ, Rai AS. Development and temporal validation of clinical prediction models for 1-year disability and pain after lumbar decompressive surgery. The Norwich Lumbar Surgery Predictor (development version). *Eur Spine J* 2023; **32**: 4210-9.

4. Debray TPA, Collins GS, Riley RD, et al. Transparent reporting of multivariable prediction models developed or validated using clustered data (TRIPOD-Cluster): explanation and elaboration. *BMJ* 2023; **380**: e071058.
