# Supplementary material for: Generalisable prediction models for outcomes after lumbar spinal stenosis surgery: a model development and external validation study
Source: eClinicalMedicine. 2026 May 28;96:103989. doi: 10.1016/j.eclinm.2026.103989 (PMC13233580; doi:10.1016/j.eclinm.2026.103989)
Supplement: Protocol [file mmc2.docx]

**Predicting disability and pain following lumbar spinal stenosis surgery: development and external validation of multivariable prediction models**

Bjørnar Berg^1^, Allan Abbott^2,3^, Henrik Hedevik^2^, Casper Friis Pedersen^4^, Martin A. Gorosito^1,5^, Henrik Lykke Joakimsen^6,7^, Per Joel Burman^7^, Karl Øyvind Mikalsen^6,7,8^, Mikkel Østerheden Andersen^3^, Tor Ingebrigtsen^6,9^, Tore Solberg^6,9^, Margreth Grotle^10^

1. Centre for Intelligent Musculoskeletal Health, Faculty of Health Sciences, Oslo Metropolitan University, Oslo, Norway

2. Unit of Physiotherapy, Department of Health, Medicine and Caring Sciences, Linköping University, Linköping, Sweden

3. Department of Orthopaedics, Linköping University Hospital, Linköping, Sweden

4. University of Southern Denmark, Center for Spine Surgery and Research, Spine center of Southern Denmark, Lillebaelt Hospital, Kolding, Denmark

5. Department of Computer Science, Oslo Metropolitan University, Oslo Norway

6. Department of Clinical Medicine, Faculty of Health Sciences, UiT The Arctic University of Norway, Tromsø, Norway

7. The Norwegian Centre for Clinical Artificial Intelligence, University Hospital of North Norway, Tromsø, Norway

8. UiT Machine Learning Group, Department of Physics and Technology, UiT the Arctic University of Norway, Tromso, Norway

9. Department of Neurosurgery and The Norwegian Registry for Spine Surgery, The University Hospital of North Norway, Tromsø, Norway

10. Department of Research and Innovation, Division of Clinical Neuroscience, Oslo University Hospital, Oslo, Norway.

**Correspondence to:**Bjørnar Berg
Centre for Intelligent Musculoskeletal Health, Faculty of Health Sciences, Oslo Metropolitan University, Oslo, Norway
Pilestredet 50, 0167 Oslo, Norway
E-mail: [bjornarb@oslomet.no](mailto:bjornarb@oslomet.no)

# AIM

To develop and validate multivariable models for predicting pain and disability 12 months after lumbar spinal stenosis surgery.

# METHODS

This will be a registry-based multicenter cohort study using prospectively collected data from three national spine registries of patients undergoing elective lumbar spinal stenosis surgery. Methods will adhere to the methodological framework proposed by the Prognosis Research Strategy (PROGRESS) group^1^ and the study will be reported consistent with the Transparent Reporting of a multivariable prediction model for Individual Prognosis or Diagnosis (TRIPOD+AI) guideline.^2^

This study is part of the AID-Spine project and has received ethical approval from the Regional Ethics Committee of the Health Region of South-East Norway (2022/371282). The Norwegian Registry for Spine Surgery (NORspine) registry protocol has been approved by the Data Protection Authority of Norway. The use of anonymized data from the Swedish Registry for Spine Surgery (SweSpine) was approved by the Regional Ethics Committee in Linköping, Sweden (2021-04914). In Denmark, the use of anonymized data from the Spine Center of Southern Denmark, registered in the Danish Registry for Spine Surgery (DaneSpine), is exempt from ethical review under Danish law. All patients in the registries are informed and have consented to their data being used for research and development purposes.

## Data sources and study population

Data from the NORspine^3^ will be used for model development and internal-external cross-validation, while external validation of the prediction models will be conducted using data from SweSpine and DaneSpine.^4,5^ NORspine has a coverage rate of 81%,^6^ and patients undergoing surgery between January 1, 2007, and December 01, 2023, will be included. SweSpine, with a coverage rate of 86%,^4^ will contribute data from patients who underwent surgery from January 1, 2016, to December 31, 2022. DaneSpine will provide data from patients enrolled at the Spine Center of Southern Denmark between August 1, 2009, and December 31, 2022, with a regional coverage rate of 96%.^7^ Eligible patients in both the development and validation cohorts will be defined in the same way: adults aged 16 years and above undergoing elective decompression surgery (with or without fusion) for lumbar spinal stenosis. Reoperation cases within 90 days will be excluded, while repeat surgeries performed more than 90 days after the index surgery will be included as new cases.

The registries follow standardized data collection procedures.^8^ Before surgery, patients complete a preoperative form including patient-reported outcomes upon surgical admission. Information regarding diagnosis, imaging findings, and comorbidity are recorded by the surgeons using a standardized form. At the 12-month follow-up, patients are asked to complete questionnaires that include the same patient-reported outcomes collected preoperatively.

## Outcomes

The primary outcome will be the Oswestry Disability Index (ODI) at the 12-month follow-up. The ODI is a ten-item score from 0 to 100, where higher scores indicate greater back-related disability.^9^ This score will be modeled both as a continuous outcome and as a binary outcome, using a cut-off of 22 points to reflect an acceptable symptom state.^10^ Back and leg pain intensity, measured using the Numeric Rating Scale (NRS, range 0-10), will be included as secondary outcomes, modeled continuously and dichotomized based on a score indicating acceptable pain level following spine surgery (3 points).^11^

## Predictors

Table 1 provides an overview of the variables available in the registries that will be considered for inclusion as predictors in the models. Only preoperative predictors will be included.

**Table 1.** Description of predictors

| **Variables (functional form)** | **Description** | **Cohort differences** | **Parameters** |
| --- | --- | --- | --- |
| Sex (binary) | Male or female |  | 1 |
| Age (continuous) | In years |  | 1-3 |
| Body mass index (continuous) | Self-reported height and weight, in kg/m^2^ |  | 1-3 |
| Smoker (binary) | Smoker or non-smoker |  | 1 |
| Work Status (categorical) | Working/Student, Retirement, Sick leave, Disability pension/work assessment allowance |  | 3 |
| Back pain duration (categorical) | Self-reported duration of neck pain: None or less than 3 months, 3 to 11 months, 12 to 24 months, More than 24 months. |  | 3 |
| Leg pain duration (categorical) | Self-reported duration of arm pain: None or less than 3 months, 3 to 11 months, 12 to 24 months, More than 24 months. |  | 3 |
| ODI (continuous) | Oswestry Disability Index score (range 0 to 100) |  | 1-3 |
| NRS back pain (continuous) | Numeric Rating Scale for back pain (range 0 to 10) |  | 1-3 |
| NRS leg pain (continuous) | Numeric Rating Scale for leg pain (range 0 to 10) |  | 1-3 |
| EQ-5D (continuous) | Health-related quality of life (5L). For patients responding to the 3L version, reverse crosswalk values were computed using the EQ-5D-5L Delvin value set (range -0.285 to 1) |  | 1-3 |
| EQ-VAS (continuous) | Self-reported health status using Visual Analogue Scale from EQ-5D (range 0 to 100) |  | 1-3 |
| Anxiety/Depression (binary) | Self-reported anxiety or depression, based on EQ-5D 5^th^ item: moderate to extreme |  | 1 |
| Previous surgery (categorical) | None, One, Two or more |  | 2 |
| Comorbidities (categorical) | Recorded by the surgeon, from a list of relevant comorbidities. Categorized as none, one, two, three or more | NORspine has a more extensive list of relevant comorbidities | 3 |
| ASA grade (categorical) | American Society of Anaesthesiologists grade recorded by the surgeon: Grade 1, Grade 2, Grade 3-5 |  | 2 |
| Analgesics use (Categorical) | Self-reported frequency due to back pain: Monthly or less frequent, Weekly, Daily. | SweSpine: Not using, Occasionally, Regularly | 2 |

## Sample size

The sample size will be fixed based on the available datasets (number of surgeries performed in the time periods); however, sample size calculations will be used to assess the adequacy of the cohorts for model development and validation. For model development,^12^ a minimum of 1052 surgical cases will be required for the continuous ODI score model, based on the inclusion of 44 predictor parameters (to allow for all predictors plus transformations), assuming an R^2^ of 0.30, a mean ODI score of 23.3, and a standard deviation of 18.2. For binary outcomes, model development will require at least 2078 cases (with 977 events), assuming an outcome prevalence of 47% (not experiencing an acceptable symptom state) and a C-statistic of 0.74.^13^

For external validation, sample size requirements will be calculated using input values from the developed models to confirm sufficient power for accurate discrimination and calibration assessment.^14^ We expect that the validation cohorts will exceed these minimum requirements for both continuous and binary outcomes. For example, with an R^2^ of 0.30, conservatively assumed perfect calibration, and an outcome standard deviation of 18.2, the minimum required sample size for continuous outcomes would be 905 cases to achieve CI widths of 0.1 for R^2^, 5 for calibration-in-the-large (considered precise given the ODI score scale), and 0.2 for the calibration slope. For binary outcomes, assuming a normally distributed linear predictor (mean=0.96, SD=1.54),^15^ outcome prevalence of 47% (acceptable symptom state), and a C-statistic of 0.74, the minimum required sample size would be 1939 cases (912 events) to target default CI widths.

## Missing data

To handle missing values in predictors and outcomes, multiple imputation by chained equations will be applied.^16^ The number of imputations will be based on the proportion of missing data to ensure stable and reliable estimates. All predictors and outcomes will be included in the imputation model. Imputations will be performed separately for the development and validation cohorts, allowing the distributions of imputed values to differ between cohorts.^17^ Model performance will be estimated in each of the imputed datasets, and Rubin’s rules will be used to pool the results,^18^ providing robust estimates of model performance across the imputed datasets.

## Statistical analyses

Descriptive characteristics will be calculated separately for the development and validation cohorts to assess sample relatedness. The same model performance estimates with 95% CI will be evaluated at both the model development and validation stages (Table 2).

**Table 2.** Performance measures

|  | **Continuous outcomes** | **Binary outcomes** |
| --- | --- | --- |
| Overall fit | Adjusted R^2^ |  |
|  | Mean Absolute Error |  |
| Calibration | Calibration-in-the-large | Calibration-in-the-large |
|  | Calibration slope | Calibration slope |
| Discrimination |  | C-statistic |

### Model development and internal-external cross-validation

We will employ two modeling approaches for both continuous and binary outcomes: regression-based models (linear and logistic) and a machine learning model (XGBoost). To address potential non-linear relationships in the data, multivariable fractional polynomials will be applied within the regression models.^19^ Clustered standard errors will be used in the regression models to account for clustering of patients within individual surgical centers. For XGBoost, hyperparameter tuning will be conducted using Bayesian optimization to determine optimal settings for training of the model, and categorical variables will be one-hot encoded.

To obtain a more realistic estimate of model performance and assess generalizability across geographical regions, we will use internal-external cross-validation (IECV).^20^ In the IECV approach, data will be divided into clusters based on Norway’s four Regional Health Authorities, with an additional cluster for private hospitals. In each cycle, four of the five clusters will form the development cohort, while the remaining cluster is reserved for validation, rotating through all clusters across five cycles. Model performance will be estimated within each region, and overall performance metrics, with 95% confidence intervals (using the Hartung-Knapp-Sidik-Jonkman variance correction), will be summarized across clusters using a random-effects meta-analysis.^21,22^

For regression model interpretation, regression coefficients or odds ratios (with 95% CIs) will be reported.

### Model validation

The developed models will be applied to the external validation cohorts to assess their performance. For regression models, the fractional polynomials identified during model development will be retained, and for XGBoost, the optimal hyperparameters defined in the development cohort will be used to prevent data leakage and ensure consistency in validation. The same model performance measures will be assessed through pooled performance estimates (Table 2). Additionally, calibration plots will be generated to compare observed versus predicted outcomes or probabilities, in addition to risk distribution plots and decision curve analysis for binary models.^23^

### Additional analyses

Heterogeneity in model performance will be explored for patients undergoing fusion surgery, by computing performance metrics separately using individual-level predictors.

# REFERENCES

1. Steyerberg EW, Moons KG, van der Windt DA, et al. Prognosis Research Strategy (PROGRESS) 3: prognostic model research. *PLoS Med*. 2013;10(2):e1001381.

2. Collins GS, Moons KGM, Dhiman P, et al. TRIPOD+AI statement: updated guidance for reporting clinical prediction models that use regression or machine learning methods. *BMJ*. 2024;385:e078378.

3. Mikkelsen E, Ingebrigtsen T, Thyrhaug AM, et al. The Norwegian registry for spine surgery (NORspine): cohort profile. *Eur Spine J*. 2023;32(11):3713-3730.

4. Fritzell P, Blom C, Löfgren H, et al. *Annual report 2023 SweSpine 25 year: follow-up of spine surgery in Sweden 1998-2022*. 2023. <https://www.swespine.se/uploads/231108_>årsrapport_swespine_english.pdf.

5. Andersen M, Nielsen M, Møller C, Wendell Rickers K, Eiskjær S. Rygkirurgi i Danmark Årsrapport 2023. <https://drks.ortopaedi.dk/wp-content/uploads/2024/06/Aarsrapport-DRKS-2023.pdf>

6. Solberg TK, Ingebrigtsen T, Olsen LR, Thyrhaug AM. Årsrapport 2022: Nasjonalt kvalitetsregister for ryggkirurgi, resultater og forbedringstiltak. *Septentrio Reports*. 2023;(1)

7. Hojmark K, Stottrup C, Carreon L, Andersen MO. Patient-reported outcome measures unbiased by loss of follow-up. Single-center study based on DaneSpine, the Danish spine surgery registry. *Eur Spine J*. 2016;25(1):282-286.

8. Lagerbäck T, Fritzell P, Hägg O, et al. Effectiveness of surgery for sciatica with disc herniation is not substantially affected by differences in surgical incidences among three countries: results from the Danish, Swedish and Norwegian spine registries. *Eur Spine J*. 2019;28(11):2562-2571.

9. Fairbank JC, Pynsent PB. The Oswestry Disability Index. *Spine (Phila Pa 1976)*. 2000;25(22):2940-52; discussion 2952.

10. van Hooff ML, Mannion AF, Staub LP, Ostelo RW, Fairbank JC. Determination of the Oswestry Disability Index score equivalent to a "satisfactory symptom state" in patients undergoing surgery for degenerative disorders of the lumbar spine-a Spine Tango registry-based study. *Spine J*. 2016;16(10):1221-1230.

11. Fekete TF, Haschtmann D, Kleinstück FS, Porchet F, Jeszenszky D, Mannion AF. What level of pain are patients happy to live with after surgery for lumbar degenerative disorders? *Spine J*. 2016;16(4 Suppl):S12-8.

12. Riley RD, Ensor J, Snell KIE, et al. Calculating the sample size required for developing a clinical prediction model. *BMJ*. 2020;368:m441.

13. Geere JH, Hunter PR, Swamy GN, Cook AJ, Rai AS. Development and temporal validation of clinical prediction models for 1-year disability and pain after lumbar decompressive surgery. The Norwich Lumbar Surgery Predictor (development version). *Eur Spine J*. 2023;32(12):4210-4219.

14. Riley RD, Snell KIE, Archer L, et al. Evaluation of clinical prediction models (part 3): calculating the sample size required for an external validation study. *BMJ*. 2024;384:e074821.

15. Berg B, Gorosito MA, Fjeld O, et al. Machine Learning Models for Predicting Disability and Pain Following Lumbar Disc Herniation Surgery. *JAMA Netw Open*. 2024;7(2):e2355024-e2355024.

16. White IR, Royston P, Wood AM. Multiple imputation using chained equations: Issues and guidance for practice. *Stat Med*. 2011;30(4):377-99.

17. Eddings W, Marchenko Y. Accounting for clustering with mi impute. 2011. <https://www.stata.com/support/faqs/statistics/clustering-and-mi-impute/>. Accessed May 1, 2023.

18. Rubin D. *Multiple Imputation for Nonresponse in Surveys*. New York: Wilson & Sons; 1987.

19. Royston P, Ambler G, Sauerbrei W. The use of fractional polynomials to model continuous risk variables in epidemiology. *Int J Epidemiol*. 1999;28(5):964-974.

20. Steyerberg EW, Harrell FE, Jr. Prediction models need appropriate internal, internal-external, and external validation. *J Clin Epidemiol*. 2016;69:245-7.

21. IntHout J, Ioannidis JPA, Borm GF. The Hartung-Knapp-Sidik-Jonkman method for random effects meta-analysis is straightforward and considerably outperforms the standard DerSimonian-Laird method. *BMC Med Res Methodol*. 2014;14(1):25.

22. de Jong VMT, Moons KGM, Eijkemans MJC, Riley RD, Debray TPA. Developing more generalizable prediction models from pooled studies and large clustered data sets. *Stat Med*. 2021;40(15):3533-3559.

23. Vickers AJ, Elkin EB. Decision curve analysis: a novel method for evaluating prediction models. *Med Decis Making*. 2006;26(6):565-74.
